# Supplementary figures and images for: Genetic drift, historic migration, and limited gene flow contributing to the subpopulation divergence in wild sea beet (Beta vulgaris ssp. maritima (L.) Arcang)
Source: PLoS One. 2024 Sep 6;19(9):e0308626. doi: 10.1371/journal.pone.0308626 (PMC11379190; doi:10.1371/journal.pone.0308626)

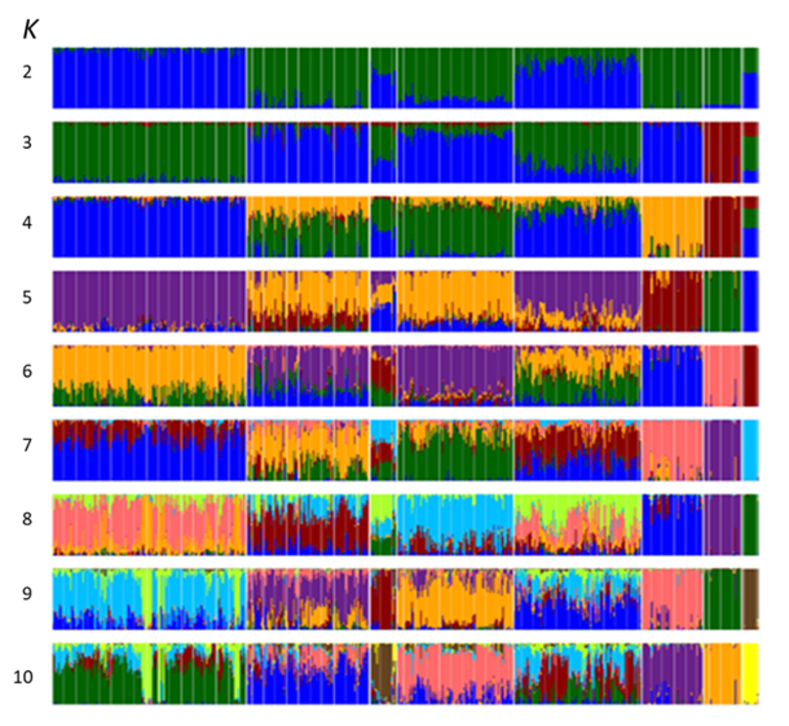

Supplement: S1 Fig — Different colors in each row represent different subpopulations. Each bar represents the estimated membership of subpopulations for a single genotype. (TIF) [file pone.0308626.s001.tif]

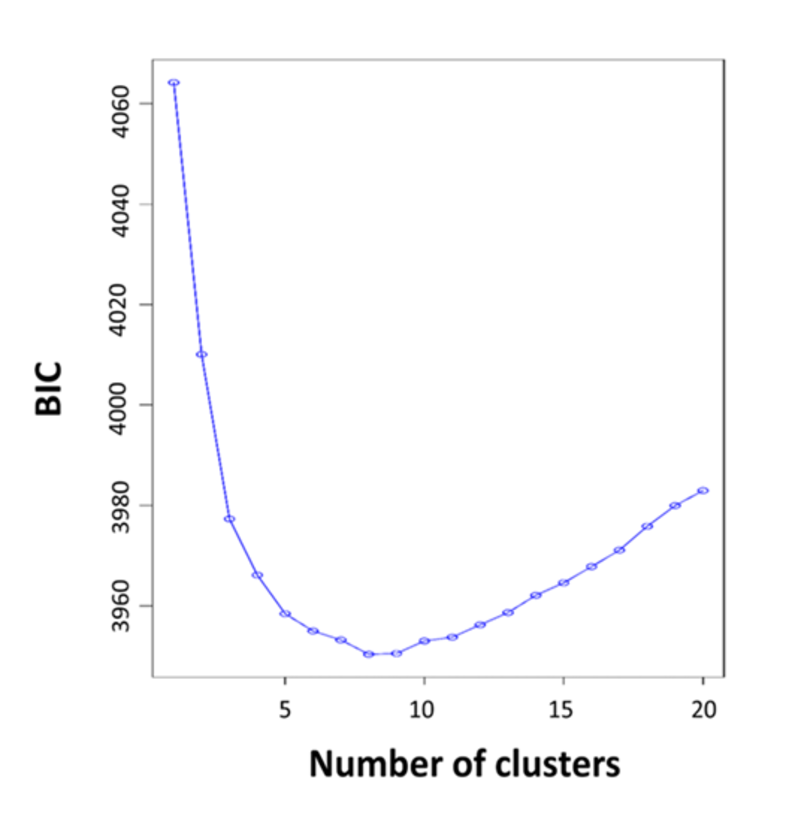

Supplement: S2 Fig — (TIF) [file pone.0308626.s002.tif]

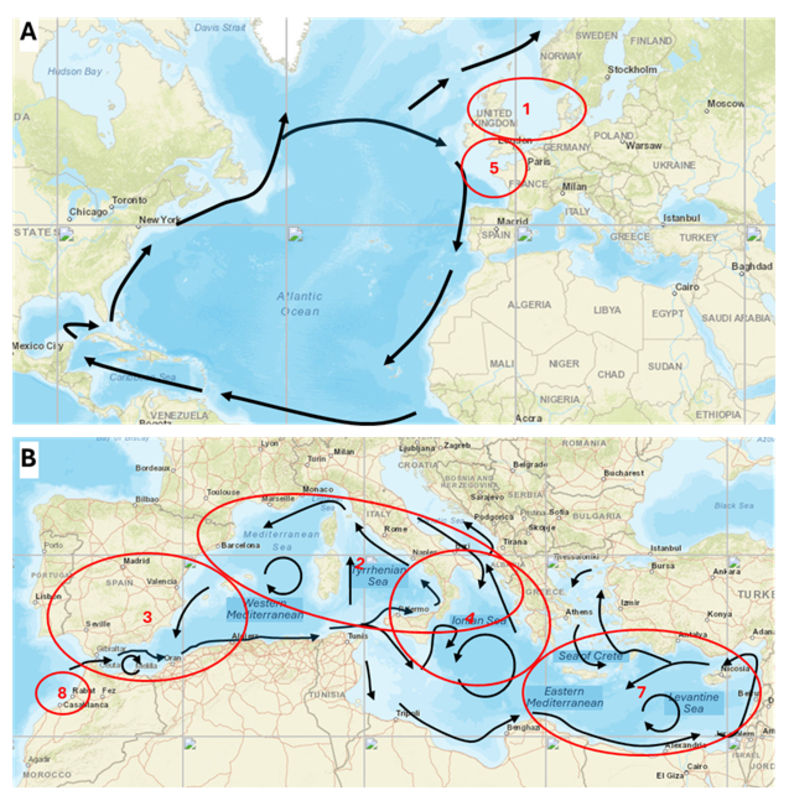

Supplement: S3 Fig — Geographical distribution of B. maritima clusters along with the marine current direction in the Atlantic Ocean (A) and the Mediterranean Sea (B). The geographic map is based on NASA world map (https://data.nasa.gov/). Black arrows indicate marine current direction of the Atlantic Ocean according to Offshore Engineering [52] and that of the Mediterranean Sea according to Pascual et al. [51]. Red circles with numbers indicate the clusters defined by DAPC (discriminant analysis of principal components) methodology with Cluster 1 contained accessions from northern and western Europe, Cluster 2 had accessions mainly from southern and western Europe, and few accessions from north America, Asia and Africa, Cluster 3 contained accessions from Morocco, Cluster 4 had accessions from southern Europe, Cluster 5 carried accessions from northern Europe (mainly from United Kingdom) and western Europe (mainly from Atlantic coast of French), Cluster 6 is corresponding to 30 cultivated beets used as the reference (it’s not shown in the picture), Cluster 7 contained accessions from Egypt and south of the Aegean Sea in southern Europe, and Cluster 8 had accessions collected from the Atlantic coast of Morrocco. (TIF) [file pone.0308626.s003.tif]
