# Supplementary material for: Genetic drift, historic migration, and limited gene flow contributing to the subpopulation divergence in wild sea beet (Beta vulgaris ssp. maritima (L.) Arcang)
Source: PLoS One. 2024 Sep 6;19(9):e0308626. doi: 10.1371/journal.pone.0308626 (PMC11379190; doi:10.1371/journal.pone.0308626)
Supplement: S1 Table — (DOCX) [file pone.0308626.s004.docx]

**S1 Table.** **List of 599 *B. maritima* accessions and 30 cultivated beet lines in eight clusters as defined by DAPC (discriminant analysis of principal components) methodology with collection locations for each accession.**

| **Line name** | **Beet type** | **Origin place collected** | **Region** | **Cluster/ Subpopulation** |
| --- | --- | --- | --- | --- |
| PI 546385 | Sea beet *(B. maritima)* | California, United States | North America | 1 |
| Ames 4219 | Sea beet *(B. maritima)* | England, United Kingdom | Northern Europe | 1 |
| PI 518300 | Sea beet *(B. maritima)* | United Kingdom | Northern Europe | 1 |
| PI 518301 | Sea beet *(B. maritima)* | England, United Kingdom | Northern Europe | 1 |
| PI 518303 | Sea beet *(B. maritima)* | England, United Kingdom | Northern Europe | 1 |
| PI 518304 | Sea beet *(B. maritima)* | England, United Kingdom | Northern Europe | 1 |
| PI 518306 | Sea beet *(B. maritima)* | England, United Kingdom | Northern Europe | 1 |
| PI 518307 | Sea beet *(B. maritima)* | England, United Kingdom | Northern Europe | 1 |
| PI 518308 | Sea beet *(B. maritima)* | England, United Kingdom | Northern Europe | 1 |
| PI 518309 | Sea beet *(B. maritima)* | England, United Kingdom | Northern Europe | 1 |
| PI 518310 | Sea beet *(B. maritima)* | England, United Kingdom | Northern Europe | 1 |
| PI 518311 | Sea beet *(B. maritima)* | England, United Kingdom | Northern Europe | 1 |
| PI 518312 | Sea beet *(B. maritima)* | England, United Kingdom | Northern Europe | 1 |
| PI 518313 | Sea beet *(B. maritima)* | England, United Kingdom | Northern Europe | 1 |
| PI 518314 | Sea beet *(B. maritima)* | England, United Kingdom | Northern Europe | 1 |
| PI 518317 | Sea beet *(B. maritima)* | England, United Kingdom | Northern Europe | 1 |
| PI 518318 | Sea beet *(B. maritima)* | England, United Kingdom | Northern Europe | 1 |
| PI 518319 | Sea beet *(B. maritima)* | England, United Kingdom | Northern Europe | 1 |
| PI 518321 | Sea beet *(B. maritima)* | England, United Kingdom | Northern Europe | 1 |
| PI 518322 | Sea beet *(B. maritima)* | England, United Kingdom | Northern Europe | 1 |
| PI 518324 | Sea beet *(B. maritima)* | England, United Kingdom | Northern Europe | 1 |
| PI 518325 | Sea beet *(B. maritima)* | England, United Kingdom | Northern Europe | 1 |
| PI 518326 | Sea beet *(B. maritima)* | England, United Kingdom | Northern Europe | 1 |
| PI 518327 | Sea beet *(B. maritima)* | England, United Kingdom | Northern Europe | 1 |
| PI 518329 | Sea beet *(B. maritima)* | United Kingdom | Northern Europe | 1 |
| PI 518330 | Sea beet *(B. maritima)* | England, United Kingdom | Northern Europe | 1 |
| PI 518331 | Sea beet *(B. maritima)* | England, United Kingdom | Northern Europe | 1 |
| PI 518333 | Sea beet *(B. maritima)* | England, United Kingdom | Northern Europe | 1 |
| PI 518334 | Sea beet *(B. maritima)* | England, United Kingdom | Northern Europe | 1 |
| PI 518336 | Sea beet *(B. maritima)* | England, United Kingdom | Northern Europe | 1 |
| PI 518337 | Sea beet *(B. maritima)* | England, United Kingdom | Northern Europe | 1 |
| PI 518338 | Sea beet *(B. maritima)* | England, United Kingdom | Northern Europe | 1 |
| PI 518339 | Sea beet *(B. maritima)* | England, United Kingdom | Northern Europe | 1 |
| PI 518340 | Sea beet *(B. maritima)* | United Kingdom | Northern Europe | 1 |
| PI 518341 | Sea beet *(B. maritima)* | United Kingdom | Northern Europe | 1 |
| PI 518342 | Sea beet *(B. maritima)* | United Kingdom | Northern Europe | 1 |
| PI 518343 | Sea beet *(B. maritima)* | England, United Kingdom | Northern Europe | 1 |
| PI 518344 | Sea beet *(B. maritima)* | England, United Kingdom | Northern Europe | 1 |
| PI 518345 | Sea beet *(B. maritima)* | England, United Kingdom | Northern Europe | 1 |
| PI 518346 | Sea beet *(B. maritima)* | England, United Kingdom | Northern Europe | 1 |
| PI 518347 | Sea beet *(B. maritima)* | England, United Kingdom | Northern Europe | 1 |
| PI 518348 | Sea beet *(B. maritima)* | England, United Kingdom | Northern Europe | 1 |
| PI 518352 | Sea beet *(B. maritima)* | England, United Kingdom | Northern Europe | 1 |
| PI 518353 | Sea beet *(B. maritima)* | England, United Kingdom | Northern Europe | 1 |
| PI 518354 | Sea beet *(B. maritima)* | England, United Kingdom | Northern Europe | 1 |
| PI 518355 | Sea beet *(B. maritima)* | England, United Kingdom | Northern Europe | 1 |
| PI 518356 | Sea beet *(B. maritima)* | England, United Kingdom | Northern Europe | 1 |
| PI 518358 | Sea beet *(B. maritima)* | Wales, United Kingdom | Northern Europe | 1 |
| PI 518359 | Sea beet *(B. maritima)* | United Kingdom | Northern Europe | 1 |
| PI 518360 | Sea beet *(B. maritima)* | Wales, United Kingdom | Northern Europe | 1 |
| PI 518361 | Sea beet *(B. maritima)* | Wales, United Kingdom | Northern Europe | 1 |
| PI 518362 | Sea beet *(B. maritima)* | United Kingdom | Northern Europe | 1 |
| PI 518363 | Sea beet *(B. maritima)* | United Kingdom | Northern Europe | 1 |
| PI 518364 | Sea beet *(B. maritima)* | United Kingdom | Northern Europe | 1 |
| PI 518365 | Sea beet *(B. maritima)* | Wales, United Kingdom | Northern Europe | 1 |
| PI 518366 | Sea beet *(B. maritima)* | United Kingdom | Northern Europe | 1 |
| PI 518367 | Sea beet *(B. maritima)* | United Kingdom | Northern Europe | 1 |
| PI 518369 | Sea beet *(B. maritima)* | Wales, United Kingdom | Northern Europe | 1 |
| PI 518370 | Sea beet *(B. maritima)* | United Kingdom | Northern Europe | 1 |
| PI 518371 | Sea beet *(B. maritima)* | United Kingdom | Northern Europe | 1 |
| PI 518372 | Sea beet *(B. maritima)* | United Kingdom | Northern Europe | 1 |
| PI 518373 | Sea beet *(B. maritima)* | Wales, United Kingdom | Northern Europe | 1 |
| PI 518374 | Sea beet *(B. maritima)* | United Kingdom | Northern Europe | 1 |
| PI 518375 | Sea beet *(B. maritima)* | United Kingdom | Northern Europe | 1 |
| PI 518377 | Sea beet *(B. maritima)* | Ireland | Northern Europe | 1 |
| PI 518378 | Sea beet *(B. maritima)* | Ireland | Northern Europe | 1 |
| PI 518379 | Sea beet *(B. maritima)* | Ireland | Northern Europe | 1 |
| PI 518381 | Sea beet *(B. maritima)* | Ireland | Northern Europe | 1 |
| PI 518382 | Sea beet *(B. maritima)* | Ireland | Northern Europe | 1 |
| PI 518383 | Sea beet *(B. maritima)* | Ireland | Northern Europe | 1 |
| PI 518384 | Sea beet *(B. maritima)* | Ireland | Northern Europe | 1 |
| PI 518387 | Sea beet *(B. maritima)* | Ireland | Northern Europe | 1 |
| PI 518388 | Sea beet *(B. maritima)* | Ireland | Northern Europe | 1 |
| PI 518389 | Sea beet *(B. maritima)* | Ireland | Northern Europe | 1 |
| PI 518390 | Sea beet *(B. maritima)* | Ireland | Northern Europe | 1 |
| PI 518392 | Sea beet *(B. maritima)* | Ireland | Northern Europe | 1 |
| PI 518394 | Sea beet *(B. maritima)* | Ireland | Northern Europe | 1 |
| PI 518395 | Sea beet *(B. maritima)* | Ireland | Northern Europe | 1 |
| PI 518396 | Sea beet *(B. maritima)* | Ireland | Northern Europe | 1 |
| PI 518397 | Sea beet *(B. maritima)* | Ireland | Northern Europe | 1 |
| PI 518398 | Sea beet *(B. maritima)* | Ireland | Northern Europe | 1 |
| PI 518399 | Sea beet *(B. maritima)* | Ireland | Northern Europe | 1 |
| PI 518400 | Sea beet *(B. maritima)* | Ireland | Northern Europe | 1 |
| PI 518401 | Sea beet *(B. maritima)* | Ireland | Northern Europe | 1 |
| PI 518402 | Sea beet *(B. maritima)* | Ireland | Northern Europe | 1 |
| PI 518403 | Sea beet *(B. maritima)* | Ireland | Northern Europe | 1 |
| PI 518408 | Sea beet *(B. maritima)* | Ireland | Northern Europe | 1 |
| PI 518411 | Sea beet *(B. maritima)* | Ireland | Northern Europe | 1 |
| PI 518413 | Sea beet *(B. maritima)* | Ireland | Northern Europe | 1 |
| PI 518414 | Sea beet *(B. maritima)* | Ireland | Northern Europe | 1 |
| PI 518415 | Sea beet *(B. maritima)* | Ireland | Northern Europe | 1 |
| PI 518416 | Sea beet *(B. maritima)* | Ireland | Northern Europe | 1 |
| PI 518418 | Sea beet *(B. maritima)* | Ireland | Northern Europe | 1 |
| PI 518420 | Sea beet *(B. maritima)* | United Kingdom | Northern Europe | 1 |
| PI 518429 | Sea beet *(B. maritima)* | England, United Kingdom | Northern Europe | 1 |
| PI 540628 | Sea beet *(B. maritima)* | Jersey Island | Northern Europe | 1 |
| PI 540629 | Sea beet *(B. maritima)* | Jersey Island | Northern Europe | 1 |
| PI 540630 | Sea beet *(B. maritima)* | United Kingdom | Northern Europe | 1 |
| PI 540631 | Sea beet *(B. maritima)* | United Kingdom | Northern Europe | 1 |
| PI 540632 | Sea beet *(B. maritima)* | United Kingdom | Northern Europe | 1 |
| PI 540633 | Sea beet *(B. maritima)* | United Kingdom | Northern Europe | 1 |
| PI 540634 | Sea beet *(B. maritima)* | United Kingdom | Northern Europe | 1 |
| PI 540668 | Sea beet *(B. maritima)* | Denmark | Northern Europe | 1 |
| PI 540669 | Sea beet *(B. maritima)* | Denmark | Northern Europe | 1 |
| PI 540670 | Sea beet *(B. maritima)* | Denmark | Northern Europe | 1 |
| PI 540671 | Sea beet *(B. maritima)* | Denmark | Northern Europe | 1 |
| PI 540672 | Sea beet *(B. maritima)* | Denmark | Northern Europe | 1 |
| PI 540674 | Sea beet *(B. maritima)* | Denmark | Northern Europe | 1 |
| PI 540675 | Sea beet *(B. maritima)* | Denmark | Northern Europe | 1 |
| PI 540676 | Sea beet *(B. maritima)* | Denmark | Northern Europe | 1 |
| PI 540677 | Sea beet *(B. maritima)* | Denmark | Northern Europe | 1 |
| PI 540678 | Sea beet *(B. maritima)* | Denmark | Northern Europe | 1 |
| PI 540679 | Sea beet *(B. maritima)* | Denmark | Northern Europe | 1 |
| PI 540680 | Sea beet *(B. maritima)* | Denmark | Northern Europe | 1 |
| PI 540682 | Sea beet *(B. maritima)* | Denmark | Northern Europe | 1 |
| PI 540684 | Sea beet *(B. maritima)* | Denmark | Northern Europe | 1 |
| PI 540685 | Sea beet *(B. maritima)* | Denmark | Northern Europe | 1 |
| PI 546397 | Sea beet *(B. maritima)* | Denmark | Northern Europe | 1 |
| PI 546402 | Sea beet *(B. maritima)* | England, United Kingdom | Northern Europe | 1 |
| PI 546403 | Sea beet *(B. maritima)* | England, United Kingdom | Northern Europe | 1 |
| PI 546409 | Sea beet *(B. maritima)* | United Kingdom | Northern Europe | 1 |
| PI 546410 | Sea beet *(B. maritima)* | United Kingdom | Northern Europe | 1 |
| PI 546411 | Sea beet *(B. maritima)* | United Kingdom | Northern Europe | 1 |
| PI 546412 | Sea beet *(B. maritima)* | Denmark | Northern Europe | 1 |
| PI 590795 | Sea beet *(B. maritima)* | Denmark | Northern Europe | 1 |
| PI 604507 | Sea beet *(B. maritima)* | United Kingdom | Northern Europe | 1 |
| PI 504233 | Sea beet *(B. maritima)* | Italy | Southern Europe | 1 |
| PI 540568 | Sea beet *(B. maritima)* | France | Western Europe | 1 |
| PI 540571 | Sea beet *(B. maritima)* | France | Western Europe | 1 |
| PI 540591 | Sea beet *(B. maritima)* | France | Western Europe | 1 |
| PI 540602 | Sea beet *(B. maritima)* | France | Western Europe | 1 |
| PI 540608 | Sea beet *(B. maritima)* | France | Western Europe | 1 |
| PI 540611 | Sea beet *(B. maritima)* | France | Western Europe | 1 |
| PI 540612 | Sea beet *(B. maritima)* | France | Western Europe | 1 |
| PI 540614 | Sea beet *(B. maritima)* | France | Western Europe | 1 |
| PI 540616 | Sea beet *(B. maritima)* | France | Western Europe | 1 |
| PI 540617 | Sea beet *(B. maritima)* | France | Western Europe | 1 |
| PI 540618 | Sea beet *(B. maritima)* | France | Western Europe | 1 |
| PI 540620 | Sea beet *(B. maritima)* | France | Western Europe | 1 |
| PI 540621 | Sea beet *(B. maritima)* | France | Western Europe | 1 |
| PI 540622 | Sea beet *(B. maritima)* | France | Western Europe | 1 |
| PI 540623 | Sea beet *(B. maritima)* | France | Western Europe | 1 |
| PI 540624 | Sea beet *(B. maritima)* | France | Western Europe | 1 |
| PI 540625 | Sea beet *(B. maritima)* | France | Western Europe | 1 |
| PI 540626 | Sea beet *(B. maritima)* | France | Western Europe | 1 |
| PI 540627 | Sea beet *(B. maritima)* | France | Western Europe | 1 |
| PI 540635 | Sea beet *(B. maritima)* | Guernsey Island | Western Europe | 1 |
| PI 540639 | Sea beet *(B. maritima)* | France | Western Europe | 1 |
| PI 540643 | Sea beet *(B. maritima)* | France | Western Europe | 1 |
| PI 540644 | Sea beet *(B. maritima)* | France | Western Europe | 1 |
| PI 540646 | Sea beet *(B. maritima)* | France | Western Europe | 1 |
| PI 540647 | Sea beet *(B. maritima)* | France | Western Europe | 1 |
| PI 540648 | Sea beet *(B. maritima)* | France | Western Europe | 1 |
| PI 540649 | Sea beet *(B. maritima)* | France | Western Europe | 1 |
| PI 540650 | Sea beet *(B. maritima)* | France | Western Europe | 1 |
| PI 540651 | Sea beet *(B. maritima)* | France | Western Europe | 1 |
| PI 540652 | Sea beet *(B. maritima)* | France | Western Europe | 1 |
| PI 540653 | Sea beet *(B. maritima)* | France | Western Europe | 1 |
| PI 540657 | Sea beet *(B. maritima)* | France | Western Europe | 1 |
| PI 540659 | Sea beet *(B. maritima)* | France | Western Europe | 1 |
| PI 540660 | Sea beet *(B. maritima)* | France | Western Europe | 1 |
| PI 540661 | Sea beet *(B. maritima)* | France | Western Europe | 1 |
| PI 540662 | Sea beet *(B. maritima)* | France | Western Europe | 1 |
| PI 540665 | Sea beet *(B. maritima)* | France | Western Europe | 1 |
| PI 540667 | Sea beet *(B. maritima)* | France | Western Europe | 1 |
| PI 540691 | Sea beet *(B. maritima)* | France | Western Europe | 1 |
| PI 540693 | Sea beet *(B. maritima)* | France | Western Europe | 1 |
| PI 540694 | Sea beet *(B. maritima)* | France | Western Europe | 1 |
| PI 540695 | Sea beet *(B. maritima)* | France | Western Europe | 1 |
| PI 540696 | Sea beet *(B. maritima)* | France | Western Europe | 1 |
| PI 540700 | Sea beet *(B. maritima)* | France | Western Europe | 1 |
| PI 546446 | Sea beet *(B. maritima)* | France | Western Europe | 1 |
| PI 546447 | Sea beet *(B. maritima)* | France | Western Europe | 1 |
| PI 562591 | Sea beet *(B. maritima)* | Maţrūḩ, Egypt | Africa | 2 |
| PI 562597 | Sea beet *(B. maritima)* | Maţrūḩ, Egypt | Africa | 2 |
| PI 562599 | Sea beet *(B. maritima)* | Maţrūḩ, Egypt | Africa | 2 |
| PI 562600 | Sea beet *(B. maritima)* | Maţrūḩ, Egypt | Africa | 2 |
| PI 562602 | Sea beet *(B. maritima)* | Egypt | Africa | 2 |
| PI 562603 | Sea beet *(B. maritima)* | Egypt | Africa | 2 |
| PI 562604 | Sea beet *(B. maritima)* | Maţrūḩ, Egypt | Africa | 2 |
| Ames 10841 | Sea beet *(B. maritima)* | India | Asia | 2 |
| Ames 19162 | Sea beet *(B. maritima)* | Georgia | Asia | 2 |
| PI 546398 | Sea beet *(B. maritima)* | Israel | Asia | 2 |
| PI 546432 | Sea beet *(B. maritima)* | India | Asia | 2 |
| PI 546387 | Sea beet *(B. maritima)* | California, United States | North America | 2 |
| PI 546388 | Sea beet *(B. maritima)* | California, United States | North America | 2 |
| PI 546389 | Sea beet *(B. maritima)* | Salinas, CA | North America | 2 |
| PI 546390 | Sea beet *(B. maritima)* | Salinas, CA | North America | 2 |
| PI 546394 | Sea beet *(B. maritima)* | Salinas, CA | North America | 2 |
| PI 546395 | Sea beet *(B. maritima)* | California, United States | North America | 2 |
| PI 590704 | Sea beet *(B. maritima)* | California, United States | North America | 2 |
| PI 590705 | Sea beet *(B. maritima)* | California, United States | North America | 2 |
| PI 590706 | Sea beet *(B. maritima)* | California, United States | North America | 2 |
| PI 590707 | Sea beet *(B. maritima)* | California, United States | North America | 2 |
| PI 628760 | Sea beet *(B. maritima)* | California, United States | North America | 2 |
| W6 51693 | Sea beet *(B. maritima)* | California, United States | North America | 2 |
| W6 51697 | Sea beet *(B. maritima)* | California, United States | North America | 2 |
| W6 51698 | Sea beet *(B. maritima)* | California, United States | North America | 2 |
| W6 51699 | Sea beet *(B. maritima)* | California, United States | North America | 2 |
| W6 51700 | Sea beet *(B. maritima)* | California, United States | North America | 2 |
| Ames 8448 | Sea beet *(B. maritima)* | Ireland | Northern Europe | 2 |
| PI 518405 | Sea beet *(B. maritima)* | Ireland | Northern Europe | 2 |
| PI 518406 | Sea beet *(B. maritima)* | Ireland | Northern Europe | 2 |
| Ames 19160 | Sea beet *(B. maritima)* | Turkey | Southern Europe | 2 |
| Ames 19161 | Sea beet *(B. maritima)* | Turkey | Southern Europe | 2 |
| Ames 4265 | Sea beet *(B. maritima)* | Turkey | Southern Europe | 2 |
| PI 198348 | Sea beet *(B. maritima)* | Spain | Southern Europe | 2 |
| PI 198431 | Sea beet *(B. maritima)* | Italy | Southern Europe | 2 |
| PI 504172 | Sea beet *(B. maritima)* | Italy | Southern Europe | 2 |
| PI 504174 | Sea beet *(B. maritima)* | Italy | Southern Europe | 2 |
| PI 504175 | Sea beet *(B. maritima)* | Italy | Southern Europe | 2 |
| PI 504176 | Sea beet *(B. maritima)* | Italy | Southern Europe | 2 |
| PI 504177 | Sea beet *(B. maritima)* | Italy | Southern Europe | 2 |
| PI 504183 | Sea beet *(B. maritima)* | Italy | Southern Europe | 2 |
| PI 504185 | Sea beet *(B. maritima)* | Italy | Southern Europe | 2 |
| PI 504186 | Sea beet *(B. maritima)* | Italy | Southern Europe | 2 |
| PI 504187 | Sea beet *(B. maritima)* | Italy | Southern Europe | 2 |
| PI 504189 | Sea beet *(B. maritima)* | Italy | Southern Europe | 2 |
| PI 504192 | Sea beet *(B. maritima)* | Italy | Southern Europe | 2 |
| PI 504193 | Sea beet *(B. maritima)* | Italy | Southern Europe | 2 |
| PI 504197 | Sea beet *(B. maritima)* | Italy | Southern Europe | 2 |
| PI 504203 | Sea beet *(B. maritima)* | Italy | Southern Europe | 2 |
| PI 504204 | Sea beet *(B. maritima)* | Italy | Southern Europe | 2 |
| PI 504205 | Sea beet *(B. maritima)* | Italy | Southern Europe | 2 |
| PI 504224 | Sea beet *(B. maritima)* | Italy | Southern Europe | 2 |
| PI 504253 | Sea beet *(B. maritima)* | Italy | Southern Europe | 2 |
| PI 504262 | Sea beet *(B. maritima)* | Italy | Southern Europe | 2 |
| PI 504264 | Sea beet *(B. maritima)* | Italy | Southern Europe | 2 |
| PI 546396 | Sea beet *(B. maritima)* | Turkey | Southern Europe | 2 |
| PI 546399 | Sea beet *(B. maritima)* | Italy | Southern Europe | 2 |
| PI 546401 | Sea beet *(B. maritima)* | Italy | Southern Europe | 2 |
| PI 546444 | Sea beet *(B. maritima)* | Greece | Southern Europe | 2 |
| PI 546510 | Sea beet *(B. maritima)* | Greece | Southern Europe | 2 |
| PI 546513 | Sea beet *(B. maritima)* | Sicilia, Italy | Southern Europe | 2 |
| PI 546520 | Sea beet *(B. maritima)* | Aegean Islands, Greece | Southern Europe | 2 |
| PI 546521 | Sea beet *(B. maritima)* | Aegean Islands, Greece | Southern Europe | 2 |
| PI 546524 | Sea beet *(B. maritima)* | Sicilia, Italy | Southern Europe | 2 |
| PI 546530 | Sea beet *(B. maritima)* | Sicily | Southern Europe | 2 |
| PI 546531 | Sea beet *(B. maritima)* | Sicily | Southern Europe | 2 |
| PI 546532 | Sea beet *(B. maritima)* | Greece | Southern Europe | 2 |
| PI 546533 | Sea beet *(B. maritima)* | Greece | Southern Europe | 2 |
| PI 546535 | Sea beet *(B. maritima)* | Central Greece, Greece | Southern Europe | 2 |
| PI 546536 | Sea beet *(B. maritima)* | Greece | Southern Europe | 2 |
| PI 546537 | Sea beet *(B. maritima)* | Central Greece, Greece | Southern Europe | 2 |
| PI 546538 | Sea beet *(B. maritima)* | Pelopónnisos, Greece | Southern Europe | 2 |
| PI 546539 | Sea beet *(B. maritima)* | Greece | Southern Europe | 2 |
| PI 604510 | Sea beet *(B. maritima)* | Sicilia, Italy | Southern Europe | 2 |
| PI 604512 | Sea beet *(B. maritima)* | Pelopónnisos, Greece | Southern Europe | 2 |
| PI 604513 | Sea beet *(B. maritima)* | Greece | Southern Europe | 2 |
| PI 604517 | Sea beet *(B. maritima)* | Greece | Southern Europe | 2 |
| PI 604518 | Sea beet *(B. maritima)* | Greece | Southern Europe | 2 |
| PI 604519 | Sea beet *(B. maritima)* | Sicilia, Italy | Southern Europe | 2 |
| PI 604520 | Sea beet *(B. maritima)* | Valenciana, Comunidad, Spain | Southern Europe | 2 |
| PI 604522 | Sea beet *(B. maritima)* | Greece | Southern Europe | 2 |
| PI 604523 | Sea beet *(B. maritima)* | Greece | Southern Europe | 2 |
| PI 604526 | Sea beet *(B. maritima)* | Madeira Islands, Portugal | Southern Europe | 2 |
| PI 604527 | Sea beet *(B. maritima)* | Illes Balears, Spain | Southern Europe | 2 |
| PI 604529 | Sea beet *(B. maritima)* | Baleares, Spain | Southern Europe | 2 |
| PI 604535 | Sea beet *(B. maritima)* | Croatia | Southern Europe | 2 |
| PI 604545 | Sea beet *(B. maritima)* | Cyprus | Southern Europe | 2 |
| PI 604551 | Sea beet *(B. maritima)* | Veneto, Italy | Southern Europe | 2 |
| W6 21681 | Sea beet *(B. maritima)* | Greece | Southern Europe | 2 |
| PI 504180 | Sea beet *(B. maritima)* | Cosica, France | Western Europe | 2 |
| PI 504181 | Sea beet *(B. maritima)* | France | Western Europe | 2 |
| PI 504265 | Sea beet *(B. maritima)* | Cosica, France | Western Europe | 2 |
| PI 504266 | Sea beet *(B. maritima)* | France | Western Europe | 2 |
| PI 504269 | Sea beet *(B. maritima)* | France | Western Europe | 2 |
| PI 504272 | Sea beet *(B. maritima)* | France | Western Europe | 2 |
| PI 504273 | Sea beet *(B. maritima)* | France | Western Europe | 2 |
| PI 504275 | Sea beet *(B. maritima)* | France | Western Europe | 2 |
| PI 504277 | Sea beet *(B. maritima)* | France | Western Europe | 2 |
| PI 504278 | Sea beet *(B. maritima)* | France | Western Europe | 2 |
| PI 504279 | Sea beet *(B. maritima)* | France | Western Europe | 2 |
| PI 504280 | Sea beet *(B. maritima)* | France | Western Europe | 2 |
| PI 540565 | Sea beet *(B. maritima)* | France | Western Europe | 2 |
| PI 540567 | Sea beet *(B. maritima)* | France | Western Europe | 2 |
| PI 540578 | Sea beet *(B. maritima)* | France | Western Europe | 2 |
| PI 540590 | Sea beet *(B. maritima)* | France | Western Europe | 2 |
| PI 540595 | Sea beet *(B. maritima)* | France | Western Europe | 2 |
| PI 546386 | Sea beet *(B. maritima)* | France | Western Europe | 2 |
| PI 546413 | Sea beet *(B. maritima)* | France | Western Europe | 2 |
| PI 604521 | Sea beet *(B. maritima)* | Germany | Western Europe | 2 |
| PI 604547 | Sea beet *(B. maritima)* | Germany | Western Europe | 2 |
| W6 44508 | Sea beet *(B. maritima)* | Morocco | Africa | 3 |
| W6 44518 | Sea beet *(B. maritima)* | Morocco | Africa | 3 |
| W6 45829 | Sea beet *(B. maritima)* | Tanger, Morocco | Africa | 3 |
| W6 45830 | Sea beet *(B. maritima)* | Tanger, Morocco | Africa | 3 |
| W6 45836 | Sea beet *(B. maritima)* | Tanger-Tétouan-Al Hoceïma, Morocco | Africa | 3 |
| W6 45837 | Sea beet *(B. maritima)* | Tanger-Tétouan-Al Hoceïma, Morocco | Africa | 3 |
| W6 45838 | Sea beet *(B. maritima)* | Morocco | Africa | 3 |
| W6 45840 | Sea beet *(B. maritima)* | L'Oriental, Morocco | Africa | 3 |
| W6 45841 | Sea beet *(B. maritima)* | L'Oriental, Morocco | Africa | 3 |
| W6 45847 | Sea beet *(B. maritima)* | Oujda, Morocco | Africa | 3 |
| W6 45850 | Sea beet *(B. maritima)* | Morocco | Africa | 3 |
| W6 45851 | Sea beet *(B. maritima)* | Morocco | Africa | 3 |
| W6 45852 | Sea beet *(B. maritima)* | L'Oriental, Morocco | Africa | 3 |
| W6 45854 | Sea beet *(B. maritima)* | Oujda, Morocco | Africa | 3 |
| W6 45855 | Sea beet *(B. maritima)* | Morocco | Africa | 3 |
| PI 546383 | Sea beet *(B. maritima)* | Salinas, CA | North America | 3 |
| PI 546393 | Sea beet *(B. maritima)* | California, United States | North America | 3 |
| PI 546515 | Sea beet *(B. maritima)* | Greece | Southern Europe | 3 |
| PI 604524 | Sea beet *(B. maritima)* | Lisboa, Portugal | Southern Europe | 3 |
| PI 604536 | Sea beet *(B. maritima)* | Murcia, Región de, Spain | Southern Europe | 3 |
| PI 604537 | Sea beet *(B. maritima)* | Portugal | Southern Europe | 3 |
| PI 604538 | Sea beet *(B. maritima)* | Portugal | Southern Europe | 3 |
| PI 604539 | Sea beet *(B. maritima)* | Portugal | Southern Europe | 3 |
| PI 604540 | Sea beet *(B. maritima)* | Andalucía, Spain | Southern Europe | 3 |
| PI 546534 | Sea beet *(B. maritima)* | Tunisia | Africa | 4 |
| PI 546391 | Sea beet *(B. maritima)* | Salinas, CA | North America | 4 |
| PI 504178 | Sea beet *(B. maritima)* | Italy | Southern Europe | 4 |
| PI 504179 | Sea beet *(B. maritima)* | Italy | Southern Europe | 4 |
| PI 504182 | Sea beet *(B. maritima)* | Italy | Southern Europe | 4 |
| PI 504184 | Sea beet *(B. maritima)* | Italy | Southern Europe | 4 |
| PI 504190 | Sea beet *(B. maritima)* | Italy | Southern Europe | 4 |
| PI 504191 | Sea beet *(B. maritima)* | Italy | Southern Europe | 4 |
| PI 504196 | Sea beet *(B. maritima)* | Italy | Southern Europe | 4 |
| PI 504198 | Sea beet *(B. maritima)* | Italy | Southern Europe | 4 |
| PI 504199 | Sea beet *(B. maritima)* | Italy | Southern Europe | 4 |
| PI 504200 | Sea beet *(B. maritima)* | Italy | Southern Europe | 4 |
| PI 504201 | Sea beet *(B. maritima)* | Italy | Southern Europe | 4 |
| PI 504202 | Sea beet *(B. maritima)* | Italy | Southern Europe | 4 |
| PI 504207 | Sea beet *(B. maritima)* | Italy | Southern Europe | 4 |
| PI 504208 | Sea beet *(B. maritima)* | Italy | Southern Europe | 4 |
| PI 504209 | Sea beet *(B. maritima)* | Italy | Southern Europe | 4 |
| PI 504210 | Sea beet *(B. maritima)* | Italy | Southern Europe | 4 |
| PI 504211 | Sea beet *(B. maritima)* | Italy | Southern Europe | 4 |
| PI 504212 | Sea beet *(B. maritima)* | Italy | Southern Europe | 4 |
| PI 504213 | Sea beet *(B. maritima)* | Italy | Southern Europe | 4 |
| PI 504214 | Sea beet *(B. maritima)* | Italy | Southern Europe | 4 |
| PI 504215 | Sea beet *(B. maritima)* | Italy | Southern Europe | 4 |
| PI 504216 | Sea beet *(B. maritima)* | Italy | Southern Europe | 4 |
| PI 504217 | Sea beet *(B. maritima)* | Italy | Southern Europe | 4 |
| PI 504220 | Sea beet *(B. maritima)* | Italy | Southern Europe | 4 |
| PI 504221 | Sea beet *(B. maritima)* | Italy | Southern Europe | 4 |
| PI 504222 | Sea beet *(B. maritima)* | Italy | Southern Europe | 4 |
| PI 504223 | Sea beet *(B. maritima)* | Italy | Southern Europe | 4 |
| PI 504225 | Sea beet *(B. maritima)* | Sardinia | Southern Europe | 4 |
| PI 504226 | Sea beet *(B. maritima)* | Italy | Southern Europe | 4 |
| PI 504227 | Sea beet *(B. maritima)* | Italy | Southern Europe | 4 |
| PI 504229 | Sea beet *(B. maritima)* | Sardinia | Southern Europe | 4 |
| PI 504231 | Sea beet *(B. maritima)* | Italy | Southern Europe | 4 |
| PI 504232 | Sea beet *(B. maritima)* | Italy | Southern Europe | 4 |
| PI 504234 | Sea beet *(B. maritima)* | Italy | Southern Europe | 4 |
| PI 504235 | Sea beet *(B. maritima)* | Italy | Southern Europe | 4 |
| PI 504236 | Sea beet *(B. maritima)* | Italy | Southern Europe | 4 |
| PI 504237 | Sea beet *(B. maritima)* | Italy | Southern Europe | 4 |
| PI 504238 | Sea beet *(B. maritima)* | Italy | Southern Europe | 4 |
| PI 504239 | Sea beet *(B. maritima)* | Italy | Southern Europe | 4 |
| PI 504240 | Sea beet *(B. maritima)* | Italy | Southern Europe | 4 |
| PI 504241 | Sea beet *(B. maritima)* | Italy | Southern Europe | 4 |
| PI 504243 | Sea beet *(B. maritima)* | Italy | Southern Europe | 4 |
| PI 504244 | Sea beet *(B. maritima)* | Italy | Southern Europe | 4 |
| PI 504246 | Sea beet *(B. maritima)* | Italy | Southern Europe | 4 |
| PI 504247 | Sea beet *(B. maritima)* | Italy | Southern Europe | 4 |
| PI 504248 | Sea beet *(B. maritima)* | Italy | Southern Europe | 4 |
| PI 504249 | Sea beet *(B. maritima)* | Italy | Southern Europe | 4 |
| PI 504250 | Sea beet *(B. maritima)* | Italy | Southern Europe | 4 |
| PI 504251 | Sea beet *(B. maritima)* | Italy | Southern Europe | 4 |
| PI 504252 | Sea beet *(B. maritima)* | Italy | Southern Europe | 4 |
| PI 504254 | Sea beet *(B. maritima)* | Italy | Southern Europe | 4 |
| PI 504255 | Sea beet *(B. maritima)* | Italy | Southern Europe | 4 |
| PI 504256 | Sea beet *(B. maritima)* | Italy | Southern Europe | 4 |
| PI 504261 | Sea beet *(B. maritima)* | Italy | Southern Europe | 4 |
| PI 546415 | Sea beet *(B. maritima)* | Greece | Southern Europe | 4 |
| PI 546416 | Sea beet *(B. maritima)* | Greece | Southern Europe | 4 |
| PI 546418 | Sea beet *(B. maritima)* | Greece | Southern Europe | 4 |
| PI 546419 | Sea beet *(B. maritima)* | Greece | Southern Europe | 4 |
| PI 546421 | Sea beet *(B. maritima)* | Greece | Southern Europe | 4 |
| PI 546422 | Sea beet *(B. maritima)* | Greece | Southern Europe | 4 |
| PI 546423 | Sea beet *(B. maritima)* | Greece | Southern Europe | 4 |
| PI 546424 | Sea beet *(B. maritima)* | Greece | Southern Europe | 4 |
| PI 546425 | Sea beet *(B. maritima)* | Italy | Southern Europe | 4 |
| PI 546426 | Sea beet *(B. maritima)* | Italy | Southern Europe | 4 |
| PI 546429 | Sea beet *(B. maritima)* | Greece | Southern Europe | 4 |
| PI 546430 | Sea beet *(B. maritima)* | Greece | Southern Europe | 4 |
| PI 546431 | Sea beet *(B. maritima)* | Greece | Southern Europe | 4 |
| PI 546442 | Sea beet *(B. maritima)* | Greece | Southern Europe | 4 |
| PI 546443 | Sea beet *(B. maritima)* | Greece | Southern Europe | 4 |
| PI 546514 | Sea beet *(B. maritima)* | Greece | Southern Europe | 4 |
| PI 546516 | Sea beet *(B. maritima)* | Greece | Southern Europe | 4 |
| PI 546517 | Sea beet *(B. maritima)* | Greece | Southern Europe | 4 |
| PI 546519 | Sea beet *(B. maritima)* | Aegean Islands, Greece | Southern Europe | 4 |
| PI 546522 | Sea beet *(B. maritima)* | Aegean Islands, Greece | Southern Europe | 4 |
| PI 546525 | Sea beet *(B. maritima)* | Sicilia, Italy | Southern Europe | 4 |
| PI 546526 | Sea beet *(B. maritima)* | Sicilia, Italy | Southern Europe | 4 |
| PI 546527 | Sea beet *(B. maritima)* | Sicilia, Italy | Southern Europe | 4 |
| PI 546528 | Sea beet *(B. maritima)* | Sicilia, Italy | Southern Europe | 4 |
| PI 546529 | Sea beet *(B. maritima)* | Sicilia, Italy | Southern Europe | 4 |
| PI 604508 | Sea beet *(B. maritima)* | Pelopónnisos, Greece | Southern Europe | 4 |
| PI 604509 | Sea beet *(B. maritima)* | Sicilia, Italy | Southern Europe | 4 |
| PI 604514 | Sea beet *(B. maritima)* | Pelopónnisos, Greece | Southern Europe | 4 |
| PI 604515 | Sea beet *(B. maritima)* | Pelopónnisos, Greece | Southern Europe | 4 |
| PI 604525 | Sea beet *(B. maritima)* | Spain | Southern Europe | 4 |
| PI 604548 | Sea beet *(B. maritima)* | Italy | Southern Europe | 4 |
| PI 604549 | Sea beet *(B. maritima)* | Italy | Southern Europe | 4 |
| PI 604550 | Sea beet *(B. maritima)* | Italy | Southern Europe | 4 |
| PI 604552 | Sea beet *(B. maritima)* | Friuli-Venezia Giulia, Italy | Southern Europe | 4 |
| PI 504268 | Sea beet *(B. maritima)* | France | Western Europe | 4 |
| PI 504271 | Sea beet *(B. maritima)* | France | Western Europe | 4 |
| PI 504276 | Sea beet *(B. maritima)* | France | Western Europe | 4 |
| PI 504281 | Sea beet *(B. maritima)* | France | Western Europe | 4 |
| PI 504282 | Sea beet *(B. maritima)* | France | Western Europe | 4 |
| PI 504283 | Sea beet *(B. maritima)* | France | Western Europe | 4 |
| PI 504284 | Sea beet *(B. maritima)* | France | Western Europe | 4 |
| PI 540561 | Sea beet *(B. maritima)* | France | Western Europe | 4 |
| PI 540562 | Sea beet *(B. maritima)* | France | Western Europe | 4 |
| PI 540563 | Sea beet *(B. maritima)* | France | Western Europe | 4 |
| PI 540564 | Sea beet *(B. maritima)* | France | Western Europe | 4 |
| PI 540566 | Sea beet *(B. maritima)* | France | Western Europe | 4 |
| PI 540574 | Sea beet *(B. maritima)* | France | Western Europe | 4 |
| PI 540692 | Sea beet *(B. maritima)* | France | Western Europe | 4 |
| PI 546392 | Sea beet *(B. maritima)* | California, United States | North America | 5 |
| PI 599351 | Sea beet *(B. maritima)* | California, United States | North America | 5 |
| A08447 | Sea beet *(B. maritima)* | Ireland | Northern Europe | 5 |
| A08448 | Sea beet *(B. maritima)* | Ireland | Northern Europe | 5 |
| Ames 8447 | Sea beet *(B. maritima)* | Ireland | Northern Europe | 5 |
| PI 518298 | Sea beet *(B. maritima)* | England, United Kingdom | Northern Europe | 5 |
| PI 518299 | Sea beet *(B. maritima)* | England, United Kingdom | Northern Europe | 5 |
| PI 518302 | Sea beet *(B. maritima)* | England, United Kingdom | Northern Europe | 5 |
| PI 518305 | Sea beet *(B. maritima)* | England, United Kingdom | Northern Europe | 5 |
| PI 518315 | Sea beet *(B. maritima)* | England, United Kingdom | Northern Europe | 5 |
| PI 518316 | Sea beet *(B. maritima)* | England, United Kingdom | Northern Europe | 5 |
| PI 518320 | Sea beet *(B. maritima)* | England, United Kingdom | Northern Europe | 5 |
| PI 518328 | Sea beet *(B. maritima)* | England, United Kingdom | Northern Europe | 5 |
| PI 518332 | Sea beet *(B. maritima)* | England, United Kingdom | Northern Europe | 5 |
| PI 518335 | Sea beet *(B. maritima)* | England, United Kingdom | Northern Europe | 5 |
| PI 518357 | Sea beet *(B. maritima)* | England, United Kingdom | Northern Europe | 5 |
| PI 518380 | Sea beet *(B. maritima)* | Ireland | Northern Europe | 5 |
| PI 518393 | Sea beet *(B. maritima)* | Ireland | Northern Europe | 5 |
| PI 518404 | Sea beet *(B. maritima)* | Ireland | Northern Europe | 5 |
| PI 518409 | Sea beet *(B. maritima)* | Ireland | Northern Europe | 5 |
| PI 518412 | Sea beet *(B. maritima)* | Ireland | Northern Europe | 5 |
| PI 518417 | Sea beet *(B. maritima)* | Ireland | Northern Europe | 5 |
| PI 518419 | Sea beet *(B. maritima)* | Ireland | Northern Europe | 5 |
| PI 518421 | Sea beet *(B. maritima)* | England, United Kingdom | Northern Europe | 5 |
| PI 518423 | Sea beet *(B. maritima)* | England, United Kingdom | Northern Europe | 5 |
| PI 518424 | Sea beet *(B. maritima)* | England, United Kingdom | Northern Europe | 5 |
| PI 518426 | Sea beet *(B. maritima)* | England, United Kingdom | Northern Europe | 5 |
| PI 518427 | Sea beet *(B. maritima)* | United Kingdom | Northern Europe | 5 |
| PI 518428 | Sea beet *(B. maritima)* | England, United Kingdom | Northern Europe | 5 |
| PI 518430 | Sea beet *(B. maritima)* | United Kingdom | Northern Europe | 5 |
| PI 518432 | Sea beet *(B. maritima)* | England, United Kingdom | Northern Europe | 5 |
| PI 518433 | Sea beet *(B. maritima)* | England, United Kingdom | Northern Europe | 5 |
| PI 518434 | Sea beet *(B. maritima)* | United Kingdom | Northern Europe | 5 |
| PI 518435 | Sea beet *(B. maritima)* | United Kingdom | Northern Europe | 5 |
| PI 518436 | Sea beet *(B. maritima)* | England, United Kingdom | Northern Europe | 5 |
| PI 518437 | Sea beet *(B. maritima)* | United Kingdom | Northern Europe | 5 |
| PI 518439 | Sea beet *(B. maritima)* | England, United Kingdom | Northern Europe | 5 |
| PI 518440 | Sea beet *(B. maritima)* | England, United Kingdom | Northern Europe | 5 |
| PI 540673 | Sea beet *(B. maritima)* | Denmark | Northern Europe | 5 |
| PI 546405 | Sea beet *(B. maritima)* | Denmark | Northern Europe | 5 |
| PI 546406 | Sea beet *(B. maritima)* | Denmark | Northern Europe | 5 |
| PI 546407 | Sea beet *(B. maritima)* | England, United Kingdom | Northern Europe | 5 |
| PI 550718 | Sea beet *(B. maritima)* | Ireland | Northern Europe | 5 |
| PI 604031 | Sea beet *(B. maritima)* | Ireland | Northern Europe | 5 |
| PI 546445 | Sea beet *(B. maritima)* | Greece | Southern Europe | 5 |
| PI 604541 | Sea beet *(B. maritima)* | Aveiro, Portugal | Southern Europe | 5 |
| PI 504274 | Sea beet *(B. maritima)* | France | Western Europe | 5 |
| PI 540569 | Sea beet *(B. maritima)* | France | Western Europe | 5 |
| PI 540572 | Sea beet *(B. maritima)* | France | Western Europe | 5 |
| PI 540573 | Sea beet *(B. maritima)* | France | Western Europe | 5 |
| PI 540575 | Sea beet *(B. maritima)* | France | Western Europe | 5 |
| PI 540576 | Sea beet *(B. maritima)* | France | Western Europe | 5 |
| PI 540579 | Sea beet *(B. maritima)* | France | Western Europe | 5 |
| PI 540580 | Sea beet *(B. maritima)* | France | Western Europe | 5 |
| PI 540581 | Sea beet *(B. maritima)* | France | Western Europe | 5 |
| PI 540582 | Sea beet *(B. maritima)* | France | Western Europe | 5 |
| PI 540583 | Sea beet *(B. maritima)* | France | Western Europe | 5 |
| PI 540584 | Sea beet *(B. maritima)* | France | Western Europe | 5 |
| PI 540585 | Sea beet *(B. maritima)* | France | Western Europe | 5 |
| PI 540586 | Sea beet *(B. maritima)* | France | Western Europe | 5 |
| PI 540587 | Sea beet *(B. maritima)* | France | Western Europe | 5 |
| PI 540588 | Sea beet *(B. maritima)* | France | Western Europe | 5 |
| PI 540589 | Sea beet *(B. maritima)* | France | Western Europe | 5 |
| PI 540592 | Sea beet *(B. maritima)* | France | Western Europe | 5 |
| PI 540593 | Sea beet *(B. maritima)* | France | Western Europe | 5 |
| PI 540594 | Sea beet *(B. maritima)* | France | Western Europe | 5 |
| PI 540596 | Sea beet *(B. maritima)* | France | Western Europe | 5 |
| PI 540597 | Sea beet *(B. maritima)* | France | Western Europe | 5 |
| PI 540598 | Sea beet *(B. maritima)* | France | Western Europe | 5 |
| PI 540599 | Sea beet *(B. maritima)* | France | Western Europe | 5 |
| PI 540600 | Sea beet *(B. maritima)* | France | Western Europe | 5 |
| PI 540601 | Sea beet *(B. maritima)* | France | Western Europe | 5 |
| PI 540603 | Sea beet *(B. maritima)* | France | Western Europe | 5 |
| PI 540604 | Sea beet *(B. maritima)* | France | Western Europe | 5 |
| PI 540605 | Sea beet *(B. maritima)* | France | Western Europe | 5 |
| PI 540606 | Sea beet *(B. maritima)* | France | Western Europe | 5 |
| PI 540607 | Sea beet *(B. maritima)* | France | Western Europe | 5 |
| PI 540609 | Sea beet *(B. maritima)* | France | Western Europe | 5 |
| PI 540610 | Sea beet *(B. maritima)* | France | Western Europe | 5 |
| PI 540613 | Sea beet *(B. maritima)* | France | Western Europe | 5 |
| PI 540615 | Sea beet *(B. maritima)* | France | Western Europe | 5 |
| PI 540619 | Sea beet *(B. maritima)* | France | Western Europe | 5 |
| PI 540636 | Sea beet *(B. maritima)* | France | Western Europe | 5 |
| PI 540638 | Sea beet *(B. maritima)* | France | Western Europe | 5 |
| PI 540640 | Sea beet *(B. maritima)* | France | Western Europe | 5 |
| PI 540641 | Sea beet *(B. maritima)* | France | Western Europe | 5 |
| PI 540642 | Sea beet *(B. maritima)* | France | Western Europe | 5 |
| PI 540645 | Sea beet *(B. maritima)* | France | Western Europe | 5 |
| PI 540654 | Sea beet *(B. maritima)* | France | Western Europe | 5 |
| PI 540655 | Sea beet *(B. maritima)* | France | Western Europe | 5 |
| PI 540656 | Sea beet *(B. maritima)* | France | Western Europe | 5 |
| PI 540658 | Sea beet *(B. maritima)* | France | Western Europe | 5 |
| PI 540663 | Sea beet *(B. maritima)* | France | Western Europe | 5 |
| PI 540664 | Sea beet *(B. maritima)* | France | Western Europe | 5 |
| PI 540666 | Sea beet *(B. maritima)* | France | Western Europe | 5 |
| PI 540687 | Sea beet *(B. maritima)* | Belgium | Western Europe | 5 |
| PI 540688 | Sea beet *(B. maritima)* | Belgium | Western Europe | 5 |
| PI 540689 | Sea beet *(B. maritima)* | Belgium | Western Europe | 5 |
| PI 540690 | Sea beet *(B. maritima)* | France | Western Europe | 5 |
| PI 540697 | Sea beet *(B. maritima)* | France | Western Europe | 5 |
| PI 540698 | Sea beet *(B. maritima)* | France | Western Europe | 5 |
| PI 540699 | Sea beet *(B. maritima)* | France | Western Europe | 5 |
| PI 540701 | Sea beet *(B. maritima)* | France | Western Europe | 5 |
| PI 540702 | Sea beet *(B. maritima)* | France | Western Europe | 5 |
| PI 546404 | Sea beet *(B. maritima)* | Netherlands | Western Europe | 5 |
| PI 604511 | Sea beet *(B. maritima)* | Hauts-de-France, France | Western Europe | 5 |
| PI 604530 | Sea beet *(B. maritima)* | Nouvelle-Aquitaine, France | Western Europe | 5 |
| PI 604531 | Sea beet *(B. maritima)* | Nouvelle-Aquitaine, France | Western Europe | 5 |
| PI 604532 | Sea beet *(B. maritima)* | Bretagne, France | Western Europe | 5 |
| PI 604533 | Sea beet *(B. maritima)* | Bretagne, France | Western Europe | 5 |
| PI 604534 | Sea beet *(B. maritima)* | Netherlands | Western Europe | 5 |
| PI 604542 | Sea beet *(B. maritima)* | Bretagne, France | Western Europe | 5 |
| PI 604543 | Sea beet *(B. maritima)* | Bretagne, France | Western Europe | 5 |
| PI 604544 | Sea beet *(B. maritima)* | Bretagne, France | Western Europe | 5 |
| W6 45825 | Sea beet *(B. maritima)* | Tanger-Tétouan-Al Hoceïma, Morocco | Africa | 6 |
| Ames 19158 | Sea beet *(B. maritima)* | Uzbekistan | Asia | 6 |
| PI 604553 | Sea beet *(B. maritima)* | China | Asia | 6 |
| PI 518323 | Sea beet *(B. maritima)* | United Kingdom | Northern Europe | 6 |
| PI 518349 | Sea beet *(B. maritima)* | United Kingdom | Northern Europe | 6 |
| PI 518351 | Sea beet *(B. maritima)* | United Kingdom | Northern Europe | 6 |
| PI 518368 | Sea beet *(B. maritima)* | United Kingdom | Northern Europe | 6 |
| PI 518376 | Sea beet *(B. maritima)* | Ireland | Northern Europe | 6 |
| PI 518385 | Sea beet *(B. maritima)* | Ireland | Northern Europe | 6 |
| PI 518386 | Sea beet *(B. maritima)* | Ireland | Northern Europe | 6 |
| PI 518391 | Sea beet *(B. maritima)* | Ireland | Northern Europe | 6 |
| PI 518407 | Sea beet *(B. maritima)* | Ireland | Northern Europe | 6 |
| PI 518431 | Sea beet *(B. maritima)* | United Kingdom | Northern Europe | 6 |
| PI 518438 | Sea beet *(B. maritima)* | United Kingdom | Northern Europe | 6 |
| PI 504245 | Sea beet *(B. maritima)* | Italy | Southern Europe | 6 |
| PI 504259 | Sea beet *(B. maritima)* | Italy | Southern Europe | 6 |
| PI 546420 | Sea beet *(B. maritima)* | Greece | Southern Europe | 6 |
| PI 546433 | Sea beet *(B. maritima)* | Turkey | Southern Europe | 6 |
| PI 546436 | Sea beet *(B. maritima)* | Greece | Southern Europe | 6 |
| PI 546511 | Sea beet *(B. maritima)* | Greece | Southern Europe | 6 |
| PI 604528 | Sea beet *(B. maritima)* | Baleares, Spain | Southern Europe | 6 |
| PI 504270 | Sea beet *(B. maritima)* | France | Western Europe | 6 |
| PI 504285 | Sea beet *(B. maritima)* | France | Western Europe | 6 |
| PI 540570 | Sea beet *(B. maritima)* | France | Western Europe | 6 |
| PI 546414 | Sea beet *(B. maritima)* | France | Western Europe | 6 |
| PI 177273 | Fodder beet | Turkey | Asia | 6 |
| PI 206407 | Fodder beet | Turkey | Asia | 6 |
| PI 222703 | Fodder beet | Iran | Asia | 6 |
| PI 518161 | Sugarbeet | Heilongjiang Sheng, China | Asia | 6 |
| PI 518164 | Sugarbeet | China | Asia | 6 |
| FC901 | Sugarbeet | Colorado, United States | North America | 6 |
| L29 | Sugarbeet | Utah, United States | North America | 6 |
| NSL 183376 | Sugarbeet | Utah, United States | North America | 6 |
| PI 590656 | Sugarbeet | Colorado, United States | North America | 6 |
| PI 590715 | Sugarbeet | California, United States | North America | 6 |
| PI 590770 | Sugarbeet | Maryland, United States | North America | 6 |
| PI 590773 | Sugarbeet | Maryland, United States | North America | 6 |
| PI 590774 | Sugarbeet | Maryland, United States | North America | 6 |
| PI 590775 | Sugarbeet | Maryland, United States | North America | 6 |
| PI 590777 | Sugarbeet | Maryland, United States | North America | 6 |
| PI 590829 | Sugarbeet | California, United States | North America | 6 |
| PI 598075 | Sugarbeet | Michigan, United States | North America | 6 |
| PI 610420 | Sugarbeet | Maryland, United States | North America | 6 |
| PI 610421 | Sugarbeet | Maryland, United States | North America | 6 |
| PI 610422 | Sugarbeet | Maryland, United States | North America | 6 |
| PI 612769 | Sugarbeet | Utah, United States | North America | 6 |
| PI 676968 | Sugarbeet | North Dakota, United States | North America | 6 |
| PI 681718 | Sugarbeet | Colorado, United States | North America | 6 |
| PI 683514 | Sugarbeet | Idaho, United States | North America | 6 |
| PI 683548 | Sugarbeet | North Dakota, United States | North America | 6 |
| SP69550 | Sugarbeet | MD, United States | North America | 6 |
| PI 232893 | Sugarbeet | Hungary | Southern Europe | 6 |
| PI 169022 | Table beet | Turkey | Asia | 6 |
| PI 173844 | Table beet | India | North America | 6 |
| PI 590593 | Table beet | Illinois, United States | North America | 6 |
| PI 562579 | Sea beet *(B. maritima)* | Maţrūḩ, Egypt | Africa | 7 |
| PI 562580 | Sea beet *(B. maritima)* | Egypt | Africa | 7 |
| PI 562581 | Sea beet *(B. maritima)* | Maţrūḩ, Egypt | Africa | 7 |
| PI 562582 | Sea beet *(B. maritima)* | Egypt | Africa | 7 |
| PI 562583 | Sea beet *(B. maritima)* | Maţrūḩ, Egypt | Africa | 7 |
| PI 562584 | Sea beet *(B. maritima)* | Maţrūḩ, Egypt | Africa | 7 |
| PI 562585 | Sea beet *(B. maritima)* | Maţrūḩ, Egypt | Africa | 7 |
| PI 562586 | Sea beet *(B. maritima)* | Maţrūḩ, Egypt | Africa | 7 |
| PI 562587 | Sea beet *(B. maritima)* | Maţrūḩ, Egypt | Africa | 7 |
| PI 562588 | Sea beet *(B. maritima)* | Maţrūḩ, Egypt | Africa | 7 |
| PI 562589 | Sea beet *(B. maritima)* | Maţrūḩ, Egypt | Africa | 7 |
| PI 562590 | Sea beet *(B. maritima)* | Maţrūḩ, Egypt | Africa | 7 |
| PI 562592 | Sea beet *(B. maritima)* | Maţrūḩ, Egypt | Africa | 7 |
| PI 562593 | Sea beet *(B. maritima)* | Maţrūḩ, Egypt | Africa | 7 |
| PI 562594 | Sea beet *(B. maritima)* | Egypt | Africa | 7 |
| PI 562595 | Sea beet *(B. maritima)* | Maţrūḩ, Egypt | Africa | 7 |
| PI 562596 | Sea beet *(B. maritima)* | Maţrūḩ, Egypt | Africa | 7 |
| PI 562598 | Sea beet *(B. maritima)* | Maţrūḩ, Egypt | Africa | 7 |
| PI 562601 | Sea beet *(B. maritima)* | Egypt | Africa | 7 |
| PI 504206 | Sea beet *(B. maritima)* | Italy | Southern Europe | 7 |
| PI 546427 | Sea beet *(B. maritima)* | Greece | Southern Europe | 7 |
| PI 546428 | Sea beet *(B. maritima)* | Greece | Southern Europe | 7 |
| PI 546434 | Sea beet *(B. maritima)* | Greece | Southern Europe | 7 |
| PI 546435 | Sea beet *(B. maritima)* | Greece | Southern Europe | 7 |
| PI 546437 | Sea beet *(B. maritima)* | Greece | Southern Europe | 7 |
| PI 546439 | Sea beet *(B. maritima)* | Turkey | Southern Europe | 7 |
| PI 546440 | Sea beet *(B. maritima)* | Turkey | Southern Europe | 7 |
| PI 546441 | Sea beet *(B. maritima)* | Greece | Southern Europe | 7 |
| PI 546508 | Sea beet *(B. maritima)* | Greece | Southern Europe | 7 |
| PI 546509 | Sea beet *(B. maritima)* | Greece | Southern Europe | 7 |
| PI 546512 | Sea beet *(B. maritima)* | Greece | Southern Europe | 7 |
| PI 546518 | Sea beet *(B. maritima)* | Aegean Islands, Greece | Southern Europe | 7 |
| PI 546523 | Sea beet *(B. maritima)* | Aegean Islands, Greece | Southern Europe | 7 |
| PI 604516 | Sea beet *(B. maritima)* | Greece | Southern Europe | 7 |
| W6 44494 | Sea beet *(B. maritima)* | Morocco | Africa | 8 |
| W6 44495 | Sea beet *(B. maritima)* | Morocco | Africa | 8 |
| W6 44496 | Sea beet *(B. maritima)* | Morocco | Africa | 8 |
| W6 44497 | Sea beet *(B. maritima)* | Morocco | Africa | 8 |
| W6 44498 | Sea beet *(B. maritima)* | Morocco | Africa | 8 |
| W6 44499 | Sea beet *(B. maritima)* | Morocco | Africa | 8 |
| W6 44500 | Sea beet *(B. maritima)* | Morocco | Africa | 8 |
| W6 44502 | Sea beet *(B. maritima)* | Morocco | Africa | 8 |
| W6 44503 | Sea beet *(B. maritima)* | Morocco | Africa | 8 |
| W6 44506 | Sea beet *(B. maritima)* | Morocco | Africa | 8 |
| W6 45821 | Sea beet *(B. maritima)* | Rabat-Sale, Morocco | Africa | 8 |
| W6 45822 | Sea beet *(B. maritima)* | Rabat-Salé-Kénitra, Morocco | Africa | 8 |
| W6 45824 | Sea beet *(B. maritima)* | Rabat-Salé-Kénitra, Morocco | Africa | 8 |
| W6 45826 | Sea beet *(B. maritima)* | Morocco | Africa | 8 |
| W6 45827 | Sea beet *(B. maritima)* | Morocco | Africa | 8 |
