# Supplementary material for: Genetic drift, historic migration, and limited gene flow contributing to the subpopulation divergence in wild sea beet (Beta vulgaris ssp. maritima (L.) Arcang)
Source: PLoS One. 2024 Sep 6;19(9):e0308626. doi: 10.1371/journal.pone.0308626 (PMC11379190; doi:10.1371/journal.pone.0308626)
Supplement: S2 Table — (DOCX) [file pone.0308626.s005.docx]

**S2 Table.** Average major allele frequencies on each chromosome of *B. maritima* across the eight clusters as defined by DAPC (discriminant analysis of principal components) methodology.

| **Chro^a^** | **Position (Mb)** | **Average major allele frequency** | | | | | | | |  | |
| --- | --- | --- | --- | --- | --- | --- | --- | --- | --- | --- | --- |
|  |  | **Cluster 1** | **Cluster 2** | **Cluster 3** | **Cluster 4** | **Cluster 5** | **Cluster 6** | **Cluster 7** | **Cluster 8** | **Variance** |  |
| 1 | 0 | 60.7 | 81.7 | 67.2 | 73.1 | 66.8 | 92.9 | 84.3 | 66.2 | 123.1 |  |
| 1 | 1 | 71.0 | 80.0 | 73.1 | 77.3 | 73.6 | 90.3 | 78.7 | 73.7 | 37.5 |  |
| 1 | 2 | 67.8 | 81.4 | 75.4 | 81.4 | 70.8 | 89.6 | 73.2 | 70.1 | 54.1 |  |
| 1 | 3 | 54.9 | 77.6 | 53.0 | 63.1 | 63.4 | 96.6 | 65.0 | 53.1 | 221.0 |  |
| 1 | 4 | 50.0 | 79.4 | 52.5 | 60.2 | 62.8 | 98.6 | 84.1 | 47.4 | 341.7 |  |
| 1 | 5 | 53.4 | 71.3 | 49.9 | 55.2 | 62.3 | 95.5 | 89.1 | 46.4 | 337.7 |  |
| 1 | 6 | 63.8 | 75.4 | 66.7 | 72.0 | 68.2 | 92.6 | 62.9 | 63.9 | 97.4 |  |
| 1 | 7 | 65.9 | 79.0 | 71.8 | 75.9 | 70.7 | 84.2 | 74.0 | 64.2 | 43.6 |  |
| 1 | 8 | 66.7 | 80.8 | 75.8 | 79.8 | 71.1 | 83.9 | 78.7 | 72.6 | 32.6 |  |
| 1 | 9 | 59.9 | 77.6 | 66.0 | 79.6 | 67.8 | 85.9 | 75.7 | 62.6 | 83.1 |  |
| 1 | 10 | 57.6 | 77.2 | 63.6 | 73.7 | 67.5 | 93.2 | 79.8 | 57.8 | 148.2 |  |
| 1 | 11 | 62.5 | 73.7 | 63.5 | 72.8 | 70.8 | 94.4 | 62.4 | 61.6 | 120.9 |  |
| 1 | 12 | 57.1 | 73.3 | 61.9 | 70.2 | 65.9 | 97.0 | 61.9 | 59.0 | 164.3 |  |
| 1 | 13 | 66.2 | 71.4 | 66.2 | 73.2 | 69.8 | 98.4 | 61.6 | 66.6 | 129.7 |  |
| 1 | 14 | 67.2 | 69.5 | 68.7 | 73.5 | 71.3 | 96.9 | 66.0 | 68.0 | 101.9 |  |
| 1 | 15 | 60.6 | 71.7 | 66.5 | 71.9 | 68.3 | 97.1 | 65.5 | 64.9 | 126.3 |  |
| 1 | 16 | 70.7 | 64.4 | 66.5 | 62.9 | 71.7 | 95.6 | 59.1 | 66.4 | 125.8 |  |
| 1 | 17 | 66.0 | 68.8 | 71.3 | 64.7 | 70.5 | 95.6 | 69.0 | 68.9 | 96.4 |  |
| 1 | 18 | 55.4 | 70.0 | 60.8 | 66.8 | 64.0 | 94.8 | 59.3 | 55.3 | 164.6 |  |
| 1 | 19 | 65.8 | 79.2 | 67.4 | 76.8 | 73.9 | 93.2 | 75.6 | 70.8 | 73.2 |  |
| 1 | 20 | 58.0 | 76.8 | 58.8 | 74.0 | 68.5 | 94.1 | 69.4 | 58.2 | 150.6 |  |
| 1 | 21 | 69.4 | 75.5 | 71.6 | 75.0 | 73.9 | 88.7 | 71.1 | 73.5 | 35.6 |  |
| 1 | 22 | 76.1 | 79.6 | 77.6 | 79.8 | 76.0 | 86.8 | 85.8 | 84.7 | 19.3 |  |
| 1 | 23 | 70.3 | 80.4 | 71.6 | 75.7 | 73.1 | 91.1 | 73.7 | 72.4 | 46.6 |  |
| 1 | 24 | 67.0 | 76.3 | 69.2 | 75.4 | 73.2 | 90.3 | 73.2 | 76.6 | 48.9 |  |
| 1 | 25 | 72.5 | 81.8 | 72.7 | 80.8 | 77.2 | 93.2 | 79.7 | 72.8 | 48.2 |  |
| 1 | 26 | 67.5 | 75.3 | 70.4 | 75.3 | 72.1 | 86.4 | 75.8 | 71.0 | 32.7 |  |
| 1 | 27 | 69.3 | 76.5 | 68.6 | 76.1 | 72.8 | 87.5 | 77.9 | 76.2 | 34.9 |  |
| 1 | 28 | 65.2 | 79.5 | 70.0 | 79.5 | 71.0 | 95.4 | 77.9 | 65.9 | 97.5 |  |
| 1 | 29 | 64.2 | 76.9 | 65.4 | 77.3 | 70.7 | 92.4 | 76.6 | 71.4 | 78.7 |  |
| 1 | 30 | 64.5 | 78.1 | 67.5 | 78.8 | 71.3 | 90.1 | 78.9 | 73.3 | 64.4 |  |
| 1 | 31 | 70.9 | 80.3 | 75.5 | 82.7 | 76.0 | 86.5 | 76.4 | 71.5 | 29.0 |  |
| 1 | 32 | 67.9 | 82.5 | 77.9 | 84.8 | 74.3 | 88.0 | 84.7 | 73.4 | 48.0 |  |
| 1 | 33 | 73.8 | 84.0 | 79.9 | 84.4 | 77.3 | 86.9 | 82.2 | 73.2 | 25.6 |  |
| 1 | 34 | 72.8 | 81.5 | 79.6 | 83.9 | 76.4 | 86.3 | 74.8 | 75.9 | 22.2 |  |
| 1 | 35 | 74.1 | 79.7 | 80.4 | 81.0 | 75.9 | 84.8 | 75.8 | 75.5 | 13.3 |  |
| 1 | 36 | 80.1 | 77.4 | 80.0 | 78.8 | 79.2 | 87.1 | 75.7 | 81.1 | 11.1 |  |
| 1 | 37 | 75.8 | 80.8 | 76.9 | 82.9 | 77.4 | 89.8 | 71.2 | 74.1 | 33.7 |  |
| 1 | 38 | 73.6 | 83.3 | 75.4 | 83.0 | 77.4 | 91.4 | 71.3 | 73.7 | 45.3 |  |
| 1 | 39 | 73.4 | 81.4 | 78.4 | 83.3 | 76.3 | 89.3 | 73.5 | 68.8 | 42.2 |  |
| 1 | 40 | 74.0 | 81.9 | 79.0 | 82.9 | 76.4 | 90.1 | 81.6 | 81.9 | 23.2 |  |
| 1 | 41 | 76.1 | 80.1 | 79.6 | 82.0 | 77.2 | 86.0 | 76.3 | 77.1 | 11.7 |  |
| 1 | 42 | 76.7 | 78.8 | 75.9 | 76.7 | 78.6 | 88.7 | 71.7 | 73.4 | 25.9 |  |
| 1 | 43 | 73.2 | 80.9 | 81.2 | 83.1 | 74.4 | 88.8 | 79.3 | 80.8 | 24.1 |  |
| 1 | 44 | 67.1 | 77.4 | 72.7 | 77.2 | 70.1 | 84.0 | 74.6 | 74.7 | 26.2 |  |
| 1 | 45 | 77.2 | 79.5 | 80.0 | 80.9 | 78.5 | 87.1 | 76.4 | 79.1 | 10.7 |  |
| 1 | 46 | 71.6 | 78.6 | 74.9 | 78.6 | 73.6 | 84.3 | 78.9 | 77.3 | 15.3 |  |
| 1 | 47 | 73.0 | 82.8 | 81.0 | 84.6 | 76.3 | 87.2 | 74.7 | 78.0 | 25.2 |  |
| 1 | 48 | 67.4 | 79.9 | 73.7 | 78.5 | 71.1 | 87.2 | 73.4 | 71.2 | 39.6 |  |
| 1 | 49 | 71.2 | 79.6 | 72.4 | 78.1 | 73.5 | 88.5 | 76.2 | 71.7 | 33.2 |  |
| 1 | 50 | 71.4 | 78.2 | 74.2 | 77.0 | 72.8 | 85.3 | 78.1 | 72.8 | 20.1 |  |
| 1 | 51 | 58.6 | 79.9 | 62.4 | 67.1 | 62.3 | 89.3 | 81.0 | 58.4 | 140.3 |  |
| 1 | 52 | 69.7 | 80.2 | 73.8 | 78.9 | 72.1 | 86.8 | 76.7 | 73.2 | 30.1 |  |
| 1 | 53 | 61.9 | 79.0 | 67.0 | 73.9 | 65.9 | 92.0 | 70.2 | 66.2 | 93.6 |  |
| 1 | 54 | 66.7 | 78.8 | 72.1 | 76.4 | 68.9 | 88.9 | 78.0 | 73.0 | 47.8 |  |
| 1 | 55 | 71.7 | 80.3 | 75.2 | 79.4 | 73.4 | 90.8 | 78.2 | 75.6 | 35.1 |  |
| 1 | 56 | 71.0 | 77.5 | 72.4 | 76.8 | 72.5 | 89.1 | 70.5 | 71.3 | 38.6 |  |
| 1 | 57 | 69.2 | 78.2 | 72.8 | 76.7 | 72.0 | 86.2 | 69.4 | 69.3 | 35.2 |  |
| 1 | 58 | 70.2 | 77.5 | 72.2 | 74.9 | 72.3 | 87.4 | 71.2 | 71.4 | 32.1 |  |
| 1 | 59 | 66.5 | 76.7 | 71.9 | 75.2 | 68.8 | 84.2 | 74.9 | 70.6 | 30.2 |  |
| 1 | 60 | 63.2 | 80.3 | 70.3 | 74.7 | 67.8 | 90.0 | 83.9 | 68.3 | 84.2 |  |
| 1 | 61 | 65.6 | 80.0 | 69.9 | 74.7 | 69.2 | 89.2 | 79.2 | 68.1 | 62.6 |  |
| 1 | 62 | 73.0 | 79.9 | 75.9 | 78.9 | 74.6 | 87.2 | 79.0 | 75.5 | 19.7 |  |
| 1 | 63 | 66.9 | 79.8 | 73.1 | 77.4 | 70.0 | 85.9 | 82.9 | 71.3 | 44.3 |  |
| 1 | 64 | 60.9 | 78.4 | 60.1 | 73.1 | 64.9 | 87.2 | 88.8 | 59.9 | 144.9 |  |
| 2 | 0 | 78.2 | 82.0 | 81.9 | 83.6 | 79.7 | 87.7 | 79.4 | 81.7 | 8.8 |  |
| 2 | 1 | 66.1 | 77.7 | 71.2 | 74.4 | 69.5 | 85.2 | 75.5 | 70.0 | 35.4 |  |
| 2 | 2 | 70.9 | 76.1 | 72.6 | 74.4 | 71.7 | 85.2 | 71.3 | 74.5 | 21.6 |  |
| 2 | 3 | 69.5 | 80.0 | 75.0 | 77.8 | 72.2 | 88.5 | 78.4 | 74.3 | 33.5 |  |
| 2 | 4 | 71.5 | 79.6 | 74.3 | 78.8 | 73.1 | 85.3 | 75.2 | 75.4 | 19.6 |  |
| 2 | 5 | 67.2 | 78.7 | 70.7 | 75.6 | 68.4 | 90.1 | 71.8 | 70.5 | 55.5 |  |
| 2 | 6 | 71.8 | 79.1 | 72.6 | 79.2 | 72.4 | 87.1 | 82.5 | 74.5 | 30.5 |  |
| 2 | 7 | 75.6 | 77.4 | 76.6 | 78.0 | 75.7 | 85.0 | 75.3 | 76.4 | 10.0 |  |
| 2 | 8 | 71.4 | 79.0 | 74.2 | 79.1 | 72.7 | 87.6 | 72.6 | 70.1 | 33.5 |  |
| 2 | 9 | 68.5 | 79.6 | 72.6 | 78.7 | 70.3 | 89.1 | 78.7 | 75.2 | 42.2 |  |
| 2 | 10 | 55.3 | 68.1 | 56.1 | 62.5 | 57.2 | 98.1 | 63.8 | 69.4 | 193.3 |  |
| 2 | 11 | 64.9 | 77.5 | 68.6 | 75.7 | 68.5 | 89.4 | 75.9 | 68.9 | 60.4 |  |
| 2 | 12 | 66.4 | 79.0 | 70.4 | 80.0 | 68.6 | 85.2 | 74.4 | 76.8 | 40.6 |  |
| 2 | 13 | 69.5 | 76.1 | 75.0 | 75.1 | 69.6 | 83.8 | 72.2 | 75.9 | 20.9 |  |
| 2 | 14 | 67.3 | 78.6 | 73.2 | 80.7 | 70.6 | 90.9 | 74.9 | 74.0 | 52.7 |  |
| 2 | 15 | 64.1 | 74.7 | 69.1 | 75.7 | 66.9 | 92.9 | 72.2 | 71.9 | 77.1 |  |
| 2 | 16 | 63.9 | 71.8 | 69.1 | 70.0 | 66.7 | 92.6 | 69.3 | 70.1 | 77.4 |  |
| 2 | 17 | 64.7 | 66.3 | 64.4 | 69.7 | 67.3 | 93.1 | 69.6 | 66.8 | 89.0 |  |
| 2 | 18 | 72.7 | 76.6 | 77.7 | 80.9 | 73.5 | 92.6 | 79.7 | 74.9 | 40.2 |  |
| 2 | 19 | 74.1 | 82.4 | 78.3 | 83.6 | 75.8 | 90.8 | 83.3 | 82.9 | 27.8 |  |
| 2 | 20 | 65.8 | 74.6 | 64.9 | 79.9 | 69.9 | 96.5 | 71.6 | 71.1 | 103.3 |  |
| 2 | 21 | 64.8 | 76.7 | 63.8 | 84.0 | 70.0 | 95.3 | 75.9 | 76.2 | 107.2 |  |
| 2 | 22 | 66.0 | 73.3 | 66.9 | 77.0 | 67.7 | 81.0 | 74.4 | 75.2 | 28.5 |  |
| 2 | 23 | 62.7 | 77.3 | 67.0 | 77.4 | 66.9 | 80.9 | 73.9 | 68.3 | 41.9 |  |
| 2 | 24 | 60.1 | 78.4 | 66.2 | 75.4 | 65.4 | 84.6 | 77.4 | 70.0 | 66.1 |  |
| 2 | 25 | 61.8 | 76.3 | 64.0 | 72.5 | 65.4 | 87.3 | 73.5 | 61.0 | 80.5 |  |
| 2 | 26 | 64.3 | 76.6 | 66.6 | 76.3 | 67.6 | 82.3 | 74.1 | 70.3 | 37.1 |  |
| 2 | 27 | 56.7 | 82.1 | 79.5 | 83.4 | 66.0 | 88.4 | 68.3 | 60.1 | 138.8 |  |
| 2 | 28 | 61.2 | 79.6 | 68.2 | 78.7 | 67.4 | 88.9 | 66.2 | 59.7 | 102.9 |  |
| 2 | 29 | 70.0 | 74.3 | 75.6 | 75.1 | 70.8 | 85.2 | 75.1 | 70.3 | 24.0 |  |
| 2 | 30 | 72.7 | 82.1 | 74.2 | 82.2 | 74.4 | 85.3 | 75.9 | 74.4 | 22.8 |  |
| 2 | 31 | 63.7 | 79.4 | 68.5 | 80.3 | 66.9 | 85.7 | 69.1 | 70.1 | 60.5 |  |
| 2 | 32 | 68.5 | 81.5 | 74.4 | 82.3 | 72.6 | 90.5 | 76.7 | 69.6 | 54.8 |  |
| 2 | 33 | 74.9 | 81.8 | 81.7 | 83.9 | 75.8 | 87.3 | 78.8 | 83.7 | 17.9 |  |
| 2 | 34 | 76.2 | 80.2 | 82.8 | 79.7 | 77.8 | 89.8 | 85.8 | 77.7 | 21.2 |  |
| 2 | 35 | 67.8 | 82.0 | 77.4 | 82.0 | 73.4 | 90.6 | 77.8 | 73.8 | 47.7 |  |
| 2 | 36 | 64.9 | 79.3 | 68.7 | 75.6 | 70.0 | 88.3 | 75.6 | 69.6 | 55.0 |  |
| 2 | 37 | 72.4 | 79.5 | 77.9 | 79.2 | 74.7 | 91.1 | 78.3 | 73.9 | 33.4 |  |
| 2 | 38 | 73.2 | 80.2 | 76.0 | 80.5 | 75.6 | 89.1 | 75.2 | 74.4 | 26.8 |  |
| 2 | 39 | 74.4 | 80.0 | 76.1 | 80.1 | 75.6 | 85.1 | 75.8 | 78.0 | 12.4 |  |
| 2 | 40 | 65.5 | 78.3 | 67.5 | 74.0 | 69.3 | 91.2 | 74.4 | 65.7 | 73.8 |  |
| 2 | 41 | 63.6 | 76.8 | 71.0 | 74.5 | 66.9 | 85.3 | 77.7 | 67.6 | 49.9 |  |
| 2 | 42 | 69.9 | 77.5 | 75.5 | 78.0 | 72.5 | 87.2 | 70.1 | 72.8 | 31.9 |  |
| 2 | 43 | 68.1 | 79.9 | 76.0 | 80.2 | 71.3 | 90.6 | 75.4 | 73.4 | 47.2 |  |
| 2 | 44 | 66.8 | 78.9 | 75.7 | 80.1 | 69.6 | 86.7 | 73.1 | 74.6 | 39.4 |  |
| 2 | 45 | 72.3 | 79.3 | 75.9 | 79.8 | 73.4 | 85.8 | 77.2 | 75.3 | 18.5 |  |
| 2 | 46 | 63.6 | 74.7 | 68.1 | 73.1 | 65.3 | 86.3 | 74.0 | 63.5 | 59.4 |  |
| 2 | 47 | 56.1 | 73.3 | 60.8 | 68.0 | 59.5 | 89.6 | 70.0 | 56.5 | 126.2 |  |
| 2 | 48 | 65.6 | 73.1 | 66.4 | 70.9 | 67.6 | 90.6 | 67.8 | 66.7 | 68.3 |  |
| 2 | 49 | 62.5 | 75.3 | 66.7 | 72.3 | 66.6 | 88.2 | 68.0 | 63.3 | 70.1 |  |
| 2 | 50 | 63.4 | 76.7 | 71.4 | 76.7 | 66.9 | 88.4 | 69.9 | 67.6 | 62.2 |  |
| 2 | 51 | 69.7 | 81.5 | 77.0 | 81.9 | 72.1 | 87.9 | 78.9 | 77.6 | 33.0 |  |
| 2 | 52 | 58.0 | 78.5 | 62.7 | 71.1 | 62.9 | 91.9 | 62.7 | 60.6 | 132.3 |  |
| 2 | 53 | 64.2 | 80.4 | 69.2 | 75.5 | 67.3 | 89.9 | 73.6 | 66.5 | 73.1 |  |
| 2 | 54 | 63.4 | 78.1 | 66.0 | 72.0 | 68.3 | 91.4 | 68.8 | 66.1 | 82.7 |  |
| 2 | 55 | 63.0 | 80.1 | 67.8 | 75.4 | 67.9 | 90.1 | 75.4 | 67.6 | 76.4 |  |
| 2 | 56 | 76.8 | 82.7 | 79.0 | 83.4 | 78.4 | 88.5 | 82.5 | 79.2 | 14.0 |  |
| 3 | 0 | 69.5 | 78.4 | 71.3 | 76.5 | 71.4 | 86.3 | 77.3 | 72.1 | 30.4 |  |
| 3 | 1 | 67.9 | 79.1 | 69.2 | 74.1 | 71.5 | 88.3 | 70.7 | 68.2 | 48.6 |  |
| 3 | 2 | 71.9 | 79.6 | 75.7 | 79.5 | 74.6 | 88.1 | 75.5 | 75.7 | 24.5 |  |
| 3 | 3 | 73.2 | 79.6 | 77.0 | 79.5 | 74.9 | 85.7 | 72.0 | 76.8 | 18.9 |  |
| 3 | 4 | 72.3 | 81.5 | 75.8 | 80.0 | 74.7 | 88.5 | 70.6 | 76.5 | 32.8 |  |
| 3 | 5 | 71.7 | 80.4 | 76.0 | 79.2 | 74.5 | 86.9 | 71.1 | 75.6 | 26.6 |  |
| 3 | 6 | 66.8 | 80.3 | 69.0 | 74.1 | 70.6 | 89.8 | 82.3 | 68.2 | 67.1 |  |
| 3 | 7 | 72.9 | 79.5 | 76.6 | 77.5 | 74.5 | 88.8 | 78.7 | 76.0 | 23.3 |  |
| 3 | 8 | 71.2 | 79.1 | 75.6 | 79.6 | 73.8 | 88.3 | 71.6 | 75.2 | 31.0 |  |
| 3 | 9 | 71.0 | 81.0 | 79.1 | 82.4 | 74.7 | 89.4 | 76.2 | 76.3 | 31.6 |  |
| 3 | 10 | 71.5 | 79.3 | 76.4 | 79.1 | 74.0 | 90.1 | 80.3 | 75.6 | 31.4 |  |
| 3 | 11 | 72.8 | 78.3 | 77.3 | 79.3 | 75.0 | 84.9 | 81.6 | 80.3 | 14.2 |  |
| 3 | 12 | 72.3 | 80.5 | 75.6 | 81.2 | 74.5 | 86.5 | 79.5 | 73.6 | 23.1 |  |
| 3 | 13 | 76.9 | 82.8 | 80.4 | 83.6 | 78.6 | 89.1 | 77.1 | 81.0 | 16.2 |  |
| 3 | 14 | 75.5 | 81.1 | 78.0 | 81.8 | 76.7 | 89.6 | 76.3 | 76.3 | 22.4 |  |
| 3 | 15 | 70.8 | 80.2 | 76.5 | 80.1 | 74.9 | 89.4 | 77.8 | 73.2 | 32.4 |  |
| 3 | 16 | 70.3 | 79.9 | 75.9 | 79.2 | 73.7 | 87.0 | 80.5 | 72.0 | 29.6 |  |
| 3 | 17 | 75.9 | 83.6 | 80.5 | 84.4 | 78.4 | 88.4 | 80.5 | 76.9 | 17.4 |  |
| 3 | 18 | 73.1 | 82.9 | 77.3 | 83.6 | 75.3 | 88.5 | 80.7 | 77.1 | 25.5 |  |
| 3 | 19 | 68.0 | 82.0 | 76.1 | 80.8 | 72.5 | 88.3 | 79.1 | 73.5 | 40.6 |  |
| 3 | 20 | 68.0 | 78.6 | 76.3 | 78.5 | 72.3 | 84.3 | 72.0 | 74.4 | 25.2 |  |
| 3 | 21 | 70.0 | 77.6 | 71.4 | 79.2 | 71.1 | 82.8 | 78.7 | 75.9 | 21.0 |  |
| 3 | 22 | 72.3 | 77.4 | 76.9 | 79.2 | 74.8 | 83.1 | 80.5 | 77.6 | 11.0 |  |
| 3 | 23 | 66.2 | 69.8 | 68.7 | 72.1 | 67.7 | 77.2 | 72.7 | 67.0 | 13.6 |  |
| 3 | 24 | 70.2 | 77.0 | 76.8 | 78.6 | 73.5 | 84.5 | 70.8 | 77.5 | 21.2 |  |
| 3 | 25 | 63.3 | 80.5 | 69.6 | 75.6 | 69.1 | 88.3 | 70.5 | 66.9 | 66.0 |  |
| 3 | 26 | 74.8 | 79.0 | 80.5 | 79.9 | 76.4 | 90.9 | 78.1 | 79.7 | 23.3 |  |
| 3 | 27 | 71.7 | 80.4 | 73.6 | 83.8 | 76.2 | 95.1 | 68.1 | 81.0 | 70.7 |  |
| 3 | 28 | 68.3 | 81.9 | 69.8 | 83.0 | 72.0 | 90.0 | 83.8 | 71.7 | 64.7 |  |
| 3 | 29 | 63.9 | 81.3 | 64.7 | 81.2 | 69.9 | 89.3 | 75.2 | 70.1 | 80.3 |  |
| 3 | 30 | 61.9 | 83.2 | 64.9 | 80.2 | 69.7 | 93.1 | 70.6 | 69.3 | 109.7 |  |
| 3 | 31 | 62.2 | 83.0 | 63.3 | 80.7 | 70.4 | 94.7 | 68.7 | 63.7 | 136.1 |  |
| 3 | 32 | 64.3 | 81.7 | 67.2 | 79.5 | 70.4 | 89.5 | 69.0 | 68.3 | 77.4 |  |
| 3 | 33 | 76.4 | 83.9 | 77.5 | 82.9 | 78.7 | 89.0 | 74.1 | 73.0 | 29.4 |  |
| 3 | 34 | 76.4 | 81.9 | 77.9 | 81.0 | 77.0 | 90.9 | 73.1 | 72.5 | 34.9 |  |
| 3 | 35 | 73.3 | 81.5 | 75.5 | 80.9 | 76.4 | 87.0 | 74.6 | 74.9 | 22.0 |  |
| 3 | 36 | 71.1 | 82.1 | 73.1 | 81.5 | 74.3 | 85.1 | 82.9 | 81.8 | 28.1 |  |
| 3 | 37 | 75.0 | 84.9 | 78.2 | 85.5 | 76.4 | 91.5 | 78.8 | 75.4 | 35.1 |  |
| 3 | 38 | 71.8 | 83.5 | 73.9 | 80.6 | 74.3 | 88.6 | 80.2 | 76.0 | 31.8 |  |
| 3 | 39 | 72.1 | 82.3 | 77.7 | 82.9 | 76.5 | 86.9 | 79.4 | 74.8 | 23.0 |  |
| 3 | 40 | 76.3 | 81.7 | 80.1 | 82.9 | 77.8 | 87.9 | 81.1 | 82.9 | 12.5 |  |
| 3 | 41 | 74.6 | 81.7 | 80.2 | 82.4 | 77.1 | 87.5 | 82.8 | 86.3 | 18.8 |  |
| 3 | 42 | 78.6 | 80.9 | 77.4 | 79.9 | 78.3 | 88.4 | 81.3 | 76.8 | 13.6 |  |
| 3 | 43 | 71.6 | 82.5 | 76.9 | 81.2 | 75.2 | 88.9 | 77.4 | 76.7 | 27.7 |  |
| 3 | 44 | 75.3 | 80.7 | 75.9 | 79.4 | 76.3 | 86.5 | 79.7 | 77.3 | 13.4 |  |
| 3 | 45 | 68.4 | 81.4 | 76.9 | 80.3 | 72.8 | 90.6 | 76.1 | 77.7 | 42.6 |  |
| 3 | 46 | 66.5 | 80.6 | 70.7 | 77.6 | 70.8 | 89.7 | 74.2 | 67.1 | 60.8 |  |
| 3 | 47 | 70.1 | 80.5 | 76.2 | 78.8 | 73.5 | 86.2 | 76.6 | 72.5 | 25.8 |  |
| 3 | 48 | 69.5 | 80.6 | 73.9 | 78.9 | 72.0 | 87.1 | 78.5 | 72.8 | 32.7 |  |
| 3 | 49 | 72.7 | 80.5 | 77.7 | 79.0 | 75.2 | 86.0 | 83.7 | 76.4 | 19.5 |  |
| 3 | 50 | 74.0 | 75.2 | 74.3 | 75.5 | 74.6 | 87.2 | 74.2 | 74.6 | 20.1 |  |
| 3 | 51 | 66.0 | 80.1 | 70.2 | 78.4 | 70.9 | 88.8 | 64.4 | 68.4 | 68.8 |  |
| 3 | 52 | 67.8 | 79.8 | 76.1 | 79.1 | 70.9 | 86.7 | 75.9 | 73.9 | 33.5 |  |
| 3 | 53 | 68.9 | 78.6 | 72.5 | 77.8 | 71.4 | 85.7 | 72.7 | 70.8 | 30.6 |  |
| 3 | 54 | 71.5 | 80.5 | 75.2 | 79.5 | 73.7 | 88.4 | 76.6 | 73.4 | 29.2 |  |
| 3 | 55 | 73.6 | 81.0 | 77.5 | 80.8 | 75.2 | 86.8 | 75.2 | 74.7 | 20.1 |  |
| 3 | 56 | 72.1 | 79.9 | 77.3 | 80.2 | 73.6 | 85.6 | 75.7 | 76.1 | 18.3 |  |
| 3 | 57 | 68.9 | 83.7 | 71.9 | 83.4 | 71.0 | 89.8 | 73.3 | 72.2 | 59.2 |  |
| 4 | 0 | 69.3 | 76.1 | 72.6 | 76.7 | 71.7 | 87.6 | 79.3 | 73.5 | 32.4 |  |
| 4 | 1 | 69.5 | 78.1 | 73.7 | 76.5 | 71.8 | 86.4 | 80.9 | 72.5 | 30.6 |  |
| 4 | 2 | 69.3 | 81.1 | 71.9 | 77.5 | 72.0 | 91.2 | 76.8 | 71.9 | 50.6 |  |
| 4 | 3 | 76.9 | 80.7 | 76.9 | 79.8 | 77.5 | 87.8 | 76.8 | 74.4 | 16.8 |  |
| 4 | 4 | 73.9 | 80.8 | 78.4 | 81.3 | 75.9 | 86.3 | 77.9 | 78.3 | 14.3 |  |
| 4 | 5 | 74.9 | 79.3 | 77.7 | 80.8 | 75.4 | 87.6 | 79.3 | 79.7 | 15.3 |  |
| 4 | 6 | 73.3 | 77.9 | 75.2 | 76.9 | 74.1 | 86.3 | 81.8 | 77.6 | 18.6 |  |
| 4 | 7 | 68.6 | 79.0 | 74.2 | 76.3 | 70.7 | 88.6 | 86.6 | 72.5 | 52.9 |  |
| 4 | 8 | 74.1 | 77.7 | 75.8 | 80.1 | 75.9 | 85.9 | 70.9 | 76.3 | 19.6 |  |
| 4 | 9 | 67.3 | 78.0 | 66.4 | 75.3 | 69.2 | 89.1 | 69.1 | 63.8 | 68.0 |  |
| 4 | 10 | 72.5 | 77.9 | 71.4 | 74.3 | 74.8 | 88.3 | 74.4 | 69.1 | 34.4 |  |
| 4 | 11 | 71.2 | 80.7 | 76.4 | 81.1 | 74.7 | 87.6 | 76.6 | 74.3 | 26.3 |  |
| 4 | 12 | 72.0 | 81.3 | 77.7 | 81.5 | 76.1 | 91.2 | 81.2 | 68.7 | 47.2 |  |
| 4 | 13 | 72.9 | 81.9 | 79.4 | 83.4 | 75.6 | 89.6 | 79.8 | 78.1 | 26.0 |  |
| 4 | 14 | 70.8 | 84.5 | 77.8 | 84.3 | 72.9 | 91.9 | 78.3 | 76.7 | 47.5 |  |
| 4 | 15 | 69.7 | 80.0 | 72.6 | 78.5 | 73.7 | 86.4 | 79.3 | 72.9 | 29.3 |  |
| 4 | 16 | 63.6 | 81.8 | 71.6 | 77.4 | 72.8 | 90.6 | 73.3 | 69.9 | 67.4 |  |
| 4 | 17 | 68.2 | 80.8 | 74.6 | 79.2 | 73.8 | 89.7 | 77.1 | 67.0 | 52.6 |  |
| 4 | 18 | 74.8 | 81.9 | 80.8 | 82.7 | 77.3 | 85.7 | 81.1 | 77.8 | 11.8 |  |
| 4 | 19 | 81.0 | 80.4 | 82.2 | 80.3 | 80.4 | 88.2 | 71.6 | 78.7 | 20.7 |  |
| 4 | 20 | 71.7 | 80.1 | 74.5 | 80.2 | 74.4 | 87.5 | 82.1 | 75.3 | 26.9 |  |
| 4 | 21 | 68.4 | 82.4 | 72.0 | 82.0 | 73.0 | 88.2 | 77.5 | 77.7 | 41.8 |  |
| 4 | 22 | 70.0 | 78.0 | 74.9 | 77.3 | 72.7 | 85.4 | 79.2 | 72.7 | 23.2 |  |
| 4 | 23 | 67.4 | 76.5 | 73.7 | 77.6 | 70.3 | 81.6 | 78.3 | 81.5 | 25.8 |  |
| 4 | 24 | 68.7 | 81.2 | 70.4 | 80.2 | 72.1 | 87.5 | 74.1 | 73.0 | 41.7 |  |
| 4 | 25 | 73.0 | 82.1 | 72.5 | 82.3 | 75.6 | 88.7 | 81.0 | 78.8 | 29.7 |  |
| 4 | 26 | 66.8 | 80.7 | 63.9 | 79.3 | 71.1 | 92.2 | 70.9 | 65.3 | 92.8 |  |
| 4 | 27 | 66.7 | 75.9 | 63.9 | 71.2 | 67.5 | 87.9 | 71.5 | 65.2 | 60.9 |  |
| 4 | 28 | 70.1 | 77.8 | 72.6 | 77.3 | 71.2 | 89.6 | 74.5 | 77.0 | 37.6 |  |
| 4 | 29 | 71.4 | 81.2 | 74.1 | 82.4 | 73.7 | 87.1 | 88.0 | 80.0 | 38.8 |  |
| 4 | 30 | 73.9 | 81.6 | 77.3 | 81.5 | 75.5 | 86.6 | 81.3 | 79.9 | 16.4 |  |
| 4 | 31 | 73.4 | 79.7 | 76.6 | 79.4 | 75.4 | 85.8 | 79.7 | 77.9 | 13.7 |  |
| 4 | 32 | 75.4 | 79.0 | 80.8 | 81.0 | 76.8 | 87.7 | 80.0 | 77.9 | 14.0 |  |
| 4 | 33 | 68.1 | 73.5 | 72.5 | 74.9 | 70.0 | 84.2 | 72.5 | 73.7 | 22.9 |  |
| 4 | 34 | 74.3 | 74.7 | 76.6 | 76.0 | 74.6 | 85.4 | 84.5 | 83.3 | 23.3 |  |
| 4 | 35 | 70.4 | 77.3 | 78.2 | 76.0 | 73.2 | 87.1 | 79.5 | 82.8 | 27.6 |  |
| 4 | 36 | 72.2 | 75.4 | 74.7 | 76.4 | 73.6 | 87.4 | 76.8 | 73.4 | 22.8 |  |
| 4 | 37 | 75.0 | 78.3 | 77.7 | 75.3 | 77.6 | 88.8 | 79.6 | 82.1 | 19.9 |  |
| 4 | 38 | 71.0 | 79.2 | 74.5 | 79.0 | 74.9 | 88.8 | 77.8 | 76.4 | 27.4 |  |
| 4 | 39 | 67.6 | 77.2 | 72.8 | 74.3 | 72.2 | 87.0 | 72.8 | 70.9 | 33.6 |  |
| 4 | 40 | 67.2 | 77.5 | 69.5 | 73.6 | 71.6 | 92.0 | 71.1 | 70.4 | 61.4 |  |
| 4 | 41 | 67.7 | 79.0 | 75.6 | 79.2 | 71.0 | 87.8 | 75.4 | 73.8 | 36.8 |  |
| 4 | 42 | 76.3 | 80.9 | 79.1 | 83.2 | 78.1 | 86.7 | 83.9 | 77.6 | 13.1 |  |
| 4 | 43 | 63.9 | 83.6 | 76.2 | 85.3 | 70.9 | 90.6 | 81.7 | 74.8 | 74.2 |  |
| 4 | 44 | 64.7 | 77.5 | 71.8 | 76.0 | 68.8 | 85.8 | 72.7 | 72.0 | 39.8 |  |
| 4 | 45 | 67.0 | 80.2 | 75.9 | 79.8 | 70.7 | 88.9 | 77.0 | 72.6 | 45.4 |  |
| 4 | 46 | 66.1 | 74.1 | 67.8 | 72.5 | 68.5 | 91.1 | 72.1 | 65.7 | 67.4 |  |
| 4 | 47 | 69.2 | 76.8 | 72.7 | 78.1 | 72.0 | 87.3 | 75.3 | 70.1 | 33.6 |  |
| 4 | 48 | 67.7 | 77.4 | 69.3 | 76.2 | 70.1 | 85.0 | 74.2 | 67.7 | 36.0 |  |
| 4 | 49 | 64.9 | 79.2 | 69.9 | 75.9 | 69.0 | 90.1 | 70.2 | 62.4 | 78.8 |  |
| 4 | 50 | 67.3 | 79.1 | 71.7 | 79.1 | 70.4 | 86.6 | 83.9 | 72.5 | 47.3 |  |
| 4 | 51 | 63.8 | 78.1 | 72.1 | 79.0 | 67.1 | 85.9 | 77.7 | 73.9 | 50.0 |  |
| 4 | 52 | 70.9 | 79.6 | 76.8 | 79.6 | 73.5 | 86.2 | 76.9 | 73.1 | 23.2 |  |
| 4 | 53 | 72.4 | 77.9 | 75.5 | 77.6 | 73.8 | 87.6 | 73.9 | 73.8 | 23.5 |  |
| 4 | 54 | 73.2 | 80.0 | 77.6 | 81.3 | 75.0 | 88.1 | 75.7 | 78.0 | 21.6 |  |
| 4 | 55 | 64.3 | 80.6 | 69.8 | 78.7 | 70.5 | 88.9 | 74.9 | 69.0 | 62.2 |  |
| 4 | 56 | 71.9 | 81.2 | 77.7 | 80.3 | 75.2 | 86.2 | 74.6 | 76.2 | 20.4 |  |
| 4 | 57 | 71.6 | 78.2 | 76.3 | 78.7 | 73.2 | 87.8 | 76.3 | 79.8 | 24.2 |  |
| 4 | 58 | 69.0 | 79.3 | 72.3 | 78.2 | 71.8 | 89.5 | 76.4 | 71.4 | 42.8 |  |
| 4 | 59 | 68.7 | 78.4 | 71.7 | 77.2 | 71.2 | 87.1 | 72.3 | 71.5 | 35.5 |  |
| 4 | 60 | 64.6 | 77.1 | 66.9 | 71.3 | 67.9 | 88.6 | 75.5 | 65.1 | 65.6 |  |
| 4 | 61 | 64.1 | 79.0 | 68.3 | 75.1 | 67.6 | 89.2 | 81.4 | 64.8 | 80.9 |  |
| 4 | 62 | 64.9 | 77.8 | 70.9 | 76.4 | 69.4 | 87.5 | 70.4 | 68.0 | 51.3 |  |
| 4 | 63 | 68.5 | 80.2 | 70.4 | 76.5 | 72.3 | 91.0 | 77.9 | 68.5 | 57.7 |  |
| 4 | 64 | 72.8 | 78.1 | 76.9 | 78.1 | 73.8 | 86.3 | 72.4 | 75.8 | 19.8 |  |
| 4 | 65 | 57.5 | 81.2 | 62.1 | 73.4 | 62.7 | 94.3 | 78.4 | 59.8 | 165.6 |  |
| 4 | 66 | 59.8 | 82.6 | 66.0 | 84.0 | 63.9 | 81.0 | 77.4 | 71.4 | 86.6 |  |
| 5 | 0 | 69.5 | 78.7 | 74.9 | 78.6 | 71.4 | 88.9 | 72.2 | 73.2 | 38.1 |  |
| 5 | 1 | 70.0 | 80.6 | 72.0 | 77.7 | 72.0 | 88.8 | 78.9 | 72.3 | 39.2 |  |
| 5 | 2 | 68.2 | 78.2 | 70.6 | 74.2 | 71.8 | 86.9 | 77.8 | 68.8 | 39.0 |  |
| 5 | 3 | 72.8 | 82.8 | 78.0 | 83.7 | 75.5 | 89.0 | 79.0 | 75.8 | 27.9 |  |
| 5 | 4 | 68.3 | 79.8 | 74.1 | 77.4 | 71.7 | 91.4 | 74.8 | 70.7 | 51.9 |  |
| 5 | 5 | 68.3 | 75.3 | 69.2 | 72.2 | 69.5 | 92.9 | 79.0 | 67.5 | 72.0 |  |
| 5 | 6 | 66.6 | 81.0 | 73.9 | 79.9 | 69.0 | 89.1 | 79.3 | 68.6 | 60.2 |  |
| 5 | 7 | 71.4 | 80.6 | 74.5 | 80.1 | 73.7 | 85.4 | 76.8 | 75.1 | 20.6 |  |
| 5 | 8 | 65.9 | 78.2 | 70.4 | 77.8 | 68.8 | 86.4 | 74.4 | 69.2 | 45.1 |  |
| 5 | 9 | 66.7 | 76.7 | 70.4 | 77.0 | 69.9 | 89.0 | 70.5 | 66.8 | 55.1 |  |
| 5 | 10 | 68.8 | 80.1 | 74.6 | 80.4 | 71.6 | 86.5 | 80.1 | 74.7 | 32.7 |  |
| 5 | 11 | 75.9 | 79.6 | 77.4 | 79.8 | 76.9 | 86.5 | 81.1 | 77.3 | 11.4 |  |
| 5 | 12 | 76.2 | 80.6 | 80.3 | 82.6 | 77.0 | 85.1 | 80.5 | 78.8 | 8.3 |  |
| 5 | 13 | 75.2 | 81.6 | 80.3 | 82.6 | 77.1 | 86.5 | 78.4 | 80.1 | 12.1 |  |
| 5 | 14 | 73.9 | 82.2 | 79.1 | 82.3 | 76.6 | 89.2 | 78.5 | 78.5 | 21.3 |  |
| 5 | 15 | 75.2 | 79.6 | 78.9 | 81.9 | 76.0 | 85.5 | 73.4 | 80.9 | 15.6 |  |
| 5 | 16 | 76.6 | 82.0 | 80.3 | 83.8 | 78.1 | 89.7 | 73.0 | 80.6 | 25.1 |  |
| 5 | 17 | 80.4 | 82.5 | 81.7 | 83.3 | 80.3 | 87.7 | 79.7 | 82.8 | 6.6 |  |
| 5 | 18 | 76.8 | 85.2 | 82.0 | 85.3 | 78.8 | 90.1 | 82.4 | 81.3 | 17.3 |  |
| 5 | 19 | 81.0 | 84.0 | 83.3 | 84.6 | 80.8 | 89.6 | 79.1 | 78.7 | 12.6 |  |
| 5 | 20 | 78.1 | 82.6 | 83.6 | 84.4 | 80.3 | 89.1 | 79.5 | 81.4 | 11.8 |  |
| 5 | 21 | 74.7 | 80.4 | 80.2 | 81.5 | 75.8 | 86.6 | 80.7 | 82.6 | 13.9 |  |
| 5 | 22 | 73.6 | 78.1 | 75.9 | 79.1 | 74.2 | 81.7 | 75.4 | 74.2 | 8.2 |  |
| 5 | 23 | 74.3 | 79.7 | 76.8 | 80.6 | 76.1 | 84.2 | 72.0 | 78.9 | 14.9 |  |
| 5 | 24 | 71.8 | 79.3 | 76.4 | 79.9 | 74.1 | 85.5 | 75.9 | 72.5 | 20.6 |  |
| 5 | 25 | 72.8 | 79.0 | 74.9 | 78.9 | 74.7 | 86.0 | 72.9 | 73.7 | 20.2 |  |
| 5 | 26 | 75.9 | 79.1 | 79.4 | 78.1 | 77.6 | 87.9 | 72.1 | 80.8 | 20.4 |  |
| 5 | 27 | 73.0 | 78.8 | 72.6 | 77.2 | 75.0 | 88.7 | 70.8 | 77.5 | 31.2 |  |
| 5 | 28 | 77.4 | 81.9 | 80.0 | 81.2 | 79.0 | 87.7 | 70.7 | 84.6 | 25.5 |  |
| 5 | 29 | 76.8 | 79.8 | 81.1 | 79.9 | 78.2 | 84.4 | 78.0 | 79.9 | 5.4 |  |
| 5 | 30 | 75.4 | 79.2 | 79.0 | 77.9 | 77.9 | 87.9 | 72.7 | 79.3 | 19.0 |  |
| 5 | 31 | 71.4 | 82.3 | 75.1 | 81.7 | 72.9 | 88.3 | 78.0 | 78.1 | 30.8 |  |
| 5 | 32 | 74.1 | 80.5 | 75.4 | 80.6 | 74.9 | 84.2 | 73.3 | 77.2 | 14.8 |  |
| 5 | 33 | 79.5 | 83.1 | 82.2 | 83.0 | 79.6 | 87.9 | 79.3 | 80.4 | 8.3 |  |
| 5 | 34 | 75.2 | 84.0 | 78.8 | 84.8 | 76.9 | 87.7 | 79.3 | 77.6 | 19.4 |  |
| 5 | 35 | 72.6 | 83.6 | 78.0 | 84.4 | 73.9 | 86.9 | 72.0 | 84.9 | 37.5 |  |
| 5 | 36 | 73.0 | 82.3 | 75.4 | 82.6 | 75.4 | 88.7 | 77.6 | 78.1 | 26.2 |  |
| 5 | 37 | 78.4 | 83.9 | 78.0 | 85.5 | 78.6 | 88.0 | 65.6 | 78.3 | 46.6 |  |
| 5 | 38 | 79.3 | 79.2 | 82.9 | 80.4 | 80.1 | 87.1 | 75.9 | 77.4 | 12.0 |  |
| 5 | 39 | 71.2 | 82.1 | 73.7 | 80.6 | 73.6 | 86.5 | 75.9 | 83.6 | 30.6 |  |
| 5 | 40 | 73.9 | 84.0 | 74.3 | 82.8 | 76.5 | 89.7 | 75.8 | 77.3 | 31.6 |  |
| 5 | 41 | 74.1 | 82.7 | 74.5 | 80.7 | 76.8 | 90.4 | 85.7 | 77.4 | 32.9 |  |
| 5 | 42 | 72.9 | 82.1 | 74.7 | 81.6 | 76.5 | 88.7 | 81.8 | 78.6 | 25.3 |  |
| 5 | 43 | 76.1 | 80.1 | 82.9 | 80.9 | 77.9 | 86.4 | 85.4 | 82.2 | 12.3 |  |
| 5 | 44 | 73.9 | 81.2 | 79.9 | 82.5 | 75.9 | 87.4 | 78.4 | 72.8 | 23.3 |  |
| 5 | 45 | 67.3 | 76.3 | 72.3 | 77.4 | 70.1 | 84.5 | 76.4 | 71.6 | 28.6 |  |
| 5 | 46 | 74.4 | 83.1 | 78.3 | 82.2 | 77.3 | 89.0 | 73.2 | 74.2 | 29.7 |  |
| 5 | 47 | 69.8 | 80.8 | 71.4 | 78.3 | 73.9 | 90.7 | 72.6 | 79.2 | 45.7 |  |
| 5 | 48 | 71.6 | 79.6 | 76.1 | 79.3 | 74.5 | 85.4 | 79.1 | 79.2 | 16.8 |  |
| 5 | 49 | 73.2 | 81.1 | 77.6 | 83.2 | 74.5 | 89.1 | 80.3 | 79.1 | 25.2 |  |
| 5 | 50 | 71.3 | 79.0 | 73.2 | 78.7 | 73.2 | 86.2 | 74.1 | 72.5 | 24.8 |  |
| 5 | 51 | 69.4 | 83.6 | 77.0 | 85.1 | 71.9 | 89.5 | 73.7 | 67.4 | 64.4 |  |
| 5 | 52 | 72.0 | 80.4 | 76.0 | 80.6 | 73.3 | 86.0 | 78.1 | 76.4 | 20.2 |  |
| 5 | 53 | 69.2 | 79.2 | 77.1 | 80.9 | 72.6 | 86.5 | 80.0 | 74.2 | 29.3 |  |
| 5 | 54 | 74.2 | 81.8 | 79.8 | 82.6 | 75.3 | 88.3 | 77.8 | 79.4 | 20.0 |  |
| 5 | 55 | 72.9 | 79.8 | 76.8 | 79.9 | 74.7 | 87.3 | 77.8 | 74.6 | 20.4 |  |
| 5 | 56 | 68.8 | 80.0 | 72.1 | 78.1 | 72.5 | 90.0 | 77.8 | 72.5 | 44.1 |  |
| 5 | 57 | 70.6 | 82.6 | 76.5 | 80.7 | 73.9 | 86.7 | 72.3 | 73.8 | 31.6 |  |
| 5 | 58 | 70.1 | 81.6 | 73.2 | 81.1 | 72.7 | 90.6 | 76.6 | 69.6 | 51.0 |  |
| 5 | 59 | 77.8 | 80.5 | 80.3 | 81.9 | 77.8 | 87.7 | 77.6 | 79.1 | 11.2 |  |
| 5 | 60 | 75.9 | 81.1 | 77.6 | 82.4 | 77.1 | 85.7 | 79.7 | 75.8 | 12.2 |  |
| 5 | 61 | 71.0 | 81.3 | 77.8 | 82.7 | 73.7 | 86.9 | 79.1 | 75.9 | 26.3 |  |
| 5 | 62 | 73.2 | 79.8 | 76.1 | 78.6 | 74.5 | 87.1 | 71.0 | 73.1 | 26.2 |  |
| 5 | 63 | 66.6 | 78.8 | 70.2 | 76.1 | 70.0 | 90.6 | 74.8 | 70.9 | 56.3 |  |
| 5 | 64 | 63.6 | 75.3 | 70.1 | 71.9 | 66.7 | 90.0 | 78.1 | 69.4 | 67.2 |  |
| 5 | 65 | 69.3 | 76.4 | 74.2 | 76.7 | 71.6 | 89.4 | 71.6 | 73.2 | 38.8 |  |
| 5 | 66 | 65.2 | 78.9 | 70.0 | 76.0 | 68.0 | 88.5 | 71.4 | 68.5 | 57.3 |  |
| 5 | 67 | 64.9 | 77.8 | 73.5 | 75.5 | 68.7 | 87.8 | 73.3 | 73.9 | 45.5 |  |
| 6 | 0 | 60.3 | 76.8 | 62.6 | 71.5 | 65.5 | 89.1 | 73.9 | 61.5 | 95.7 |  |
| 6 | 1 | 61.1 | 78.3 | 62.0 | 67.9 | 66.8 | 93.1 | 73.5 | 59.0 | 127.9 |  |
| 6 | 2 | 66.2 | 78.6 | 71.2 | 75.8 | 68.6 | 88.4 | 79.4 | 70.4 | 52.2 |  |
| 6 | 3 | 60.7 | 80.3 | 66.7 | 74.7 | 65.0 | 91.1 | 80.6 | 64.9 | 109.2 |  |
| 6 | 4 | 68.2 | 77.9 | 73.8 | 78.2 | 69.6 | 86.8 | 78.3 | 75.3 | 33.9 |  |
| 6 | 5 | 64.3 | 79.4 | 69.4 | 76.6 | 66.8 | 87.2 | 73.0 | 67.0 | 59.9 |  |
| 6 | 6 | 70.7 | 78.9 | 71.2 | 76.9 | 72.0 | 87.2 | 78.8 | 66.6 | 41.9 |  |
| 6 | 7 | 71.9 | 79.0 | 75.0 | 79.5 | 73.2 | 86.5 | 74.9 | 74.9 | 22.1 |  |
| 6 | 8 | 66.9 | 77.1 | 71.1 | 75.0 | 68.8 | 89.5 | 69.3 | 71.4 | 51.8 |  |
| 6 | 9 | 70.9 | 79.3 | 74.1 | 77.2 | 72.2 | 90.5 | 77.2 | 71.0 | 41.6 |  |
| 6 | 10 | 69.8 | 78.9 | 74.3 | 76.6 | 71.4 | 87.6 | 75.7 | 72.5 | 31.2 |  |
| 6 | 11 | 65.1 | 74.3 | 71.7 | 73.9 | 67.7 | 86.0 | 74.7 | 70.6 | 38.7 |  |
| 6 | 12 | 68.6 | 78.6 | 74.2 | 77.0 | 70.9 | 87.5 | 72.0 | 73.4 | 34.4 |  |
| 6 | 13 | 77.1 | 81.3 | 85.0 | 84.9 | 76.3 | 87.0 | 77.9 | 79.7 | 16.5 |  |
| 6 | 14 | 72.1 | 79.7 | 77.8 | 79.5 | 73.9 | 86.0 | 78.8 | 75.6 | 18.1 |  |
| 6 | 15 | 72.3 | 81.1 | 74.8 | 78.7 | 73.9 | 91.1 | 76.4 | 75.0 | 36.1 |  |
| 6 | 16 | 74.0 | 81.6 | 79.5 | 82.0 | 75.9 | 88.6 | 77.9 | 79.1 | 19.7 |  |
| 6 | 17 | 75.4 | 80.9 | 80.1 | 83.2 | 76.7 | 87.4 | 80.5 | 72.3 | 22.3 |  |
| 6 | 18 | 70.8 | 80.1 | 76.0 | 78.6 | 73.2 | 88.4 | 75.9 | 73.9 | 29.6 |  |
| 6 | 19 | 75.6 | 78.8 | 81.2 | 79.3 | 75.9 | 83.5 | 80.6 | 83.8 | 9.5 |  |
| 6 | 20 | 74.8 | 78.5 | 77.5 | 79.0 | 76.0 | 83.3 | 77.0 | 78.3 | 6.4 |  |
| 6 | 21 | 72.6 | 81.1 | 74.8 | 79.5 | 74.4 | 90.4 | 74.5 | 73.0 | 36.3 |  |
| 6 | 22 | 63.1 | 82.5 | 69.1 | 81.3 | 68.2 | 90.0 | 78.5 | 62.7 | 100.4 |  |
| 6 | 23 | 64.4 | 78.1 | 68.3 | 77.2 | 68.4 | 89.6 | 71.9 | 70.4 | 63.4 |  |
| 6 | 24 | 75.4 | 79.3 | 79.4 | 78.9 | 76.0 | 87.6 | 78.3 | 78.7 | 13.7 |  |
| 6 | 25 | 80.0 | 81.6 | 81.9 | 80.8 | 80.2 | 85.2 | 79.2 | 80.2 | 3.5 |  |
| 6 | 26 | 64.2 | 84.0 | 66.2 | 85.0 | 68.1 | 87.8 | 70.5 | 68.8 | 91.5 |  |
| 6 | 27 | 66.6 | 81.0 | 71.9 | 80.9 | 68.5 | 87.1 | 75.3 | 70.2 | 51.7 |  |
| 6 | 28 | 75.0 | 80.1 | 79.0 | 79.3 | 74.6 | 89.1 | 76.0 | 67.0 | 39.2 |  |
| 6 | 29 | 70.6 | 79.5 | 70.3 | 79.8 | 72.7 | 84.0 | 79.3 | 76.2 | 24.7 |  |
| 6 | 30 | 75.7 | 80.9 | 78.0 | 82.0 | 76.8 | 87.3 | 83.1 | 80.0 | 14.1 |  |
| 6 | 31 | 82.8 | 80.6 | 81.3 | 80.9 | 81.1 | 85.4 | 75.2 | 78.7 | 8.8 |  |
| 6 | 32 | 75.8 | 79.9 | 78.4 | 80.8 | 75.7 | 83.8 | 78.6 | 79.2 | 7.1 |  |
| 6 | 33 | 74.9 | 79.8 | 76.5 | 80.2 | 75.6 | 84.6 | 75.4 | 84.7 | 16.3 |  |
| 6 | 34 | 70.6 | 78.1 | 71.6 | 77.0 | 72.4 | 83.2 | 81.9 | 73.0 | 23.3 |  |
| 6 | 35 | 73.7 | 80.1 | 77.6 | 80.3 | 75.5 | 85.2 | 83.5 | 80.2 | 14.7 |  |
| 6 | 36 | 72.4 | 82.2 | 76.7 | 83.7 | 74.8 | 87.1 | 74.3 | 73.3 | 30.5 |  |
| 6 | 37 | 77.7 | 79.1 | 81.4 | 81.3 | 77.7 | 85.5 | 79.3 | 82.7 | 7.2 |  |
| 6 | 38 | 75.8 | 76.7 | 78.8 | 75.8 | 75.7 | 87.8 | 71.2 | 77.7 | 22.5 |  |
| 6 | 39 | 77.9 | 77.0 | 79.4 | 77.9 | 77.5 | 80.8 | 73.8 | 81.3 | 5.6 |  |
| 6 | 40 | 80.4 | 77.5 | 82.2 | 80.4 | 79.8 | 80.8 | 77.4 | 86.3 | 8.1 |  |
| 6 | 41 | 83.7 | 82.1 | 87.4 | 84.5 | 83.1 | 88.6 | 71.4 | 84.0 | 27.1 |  |
| 6 | 42 | 81.4 | 80.1 | 86.6 | 82.8 | 80.3 | 89.3 | 71.3 | 81.8 | 27.8 |  |
| 6 | 43 | 83.6 | 83.4 | 86.9 | 84.6 | 82.8 | 88.6 | 69.7 | 82.6 | 32.3 |  |
| 6 | 44 | 83.0 | 81.0 | 88.9 | 83.1 | 82.3 | 85.8 | 77.0 | 85.8 | 12.9 |  |
| 6 | 45 | 82.1 | 78.2 | 87.9 | 79.5 | 81.8 | 87.9 | 84.4 | 84.4 | 12.7 |  |
| 6 | 46 | 80.7 | 82.1 | 81.7 | 81.9 | 82.1 | 92.2 | 79.2 | 82.2 | 15.6 |  |
| 6 | 47 | 76.6 | 81.1 | 78.0 | 81.2 | 77.0 | 86.3 | 86.5 | 81.6 | 14.8 |  |
| 6 | 48 | 76.4 | 78.1 | 80.5 | 77.6 | 77.2 | 86.7 | 79.4 | 81.9 | 11.4 |  |
| 6 | 49 | 79.1 | 82.8 | 84.5 | 83.5 | 79.7 | 89.7 | 80.1 | 86.5 | 13.4 |  |
| 6 | 50 | 84.7 | 84.7 | 87.2 | 87.5 | 84.3 | 89.8 | 78.8 | 85.9 | 10.4 |  |
| 6 | 51 | 75.7 | 79.4 | 80.8 | 80.9 | 76.1 | 89.1 | 78.4 | 76.3 | 18.9 |  |
| 6 | 52 | 77.4 | 79.2 | 75.7 | 78.9 | 78.5 | 84.7 | 79.4 | 75.4 | 8.3 |  |
| 6 | 53 | 70.9 | 82.8 | 73.0 | 82.0 | 72.9 | 88.4 | 72.4 | 74.1 | 41.3 |  |
| 6 | 54 | 71.8 | 79.4 | 74.2 | 77.8 | 74.2 | 85.3 | 75.3 | 72.8 | 19.3 |  |
| 6 | 55 | 68.5 | 81.1 | 71.7 | 79.7 | 71.7 | 85.5 | 75.3 | 67.5 | 41.4 |  |
| 6 | 56 | 72.4 | 78.8 | 72.6 | 76.9 | 74.2 | 84.0 | 77.1 | 76.5 | 14.2 |  |
| 6 | 57 | 69.3 | 79.3 | 72.8 | 75.8 | 73.5 | 86.6 | 75.9 | 67.3 | 35.9 |  |
| 6 | 58 | 67.8 | 83.7 | 72.8 | 83.5 | 72.4 | 89.3 | 78.1 | 69.0 | 61.0 |  |
| 6 | 59 | 75.4 | 80.0 | 81.4 | 80.6 | 77.5 | 89.6 | 79.7 | 83.5 | 18.2 |  |
| 6 | 60 | 80.1 | 82.6 | 79.3 | 82.5 | 80.9 | 88.1 | 82.4 | 82.9 | 7.2 |  |
| 6 | 61 | 72.9 | 81.4 | 75.6 | 80.0 | 74.5 | 86.2 | 72.7 | 73.5 | 24.1 |  |
| 6 | 62 | 76.4 | 80.5 | 74.4 | 81.3 | 76.6 | 81.9 | 75.9 | 77.8 | 7.6 |  |
| 6 | 63 | 74.3 | 80.0 | 77.2 | 79.5 | 75.5 | 85.6 | 75.5 | 75.6 | 13.8 |  |
| 6 | 64 | 71.6 | 80.7 | 76.8 | 79.3 | 73.1 | 90.4 | 78.4 | 72.8 | 36.8 |  |
| 6 | 65 | 58.7 | 72.0 | 60.1 | 66.1 | 60.2 | 94.0 | 70.0 | 59.7 | 139.7 |  |
| 6 | 66 | 67.6 | 73.0 | 68.7 | 71.5 | 69.7 | 88.8 | 86.3 | 67.3 | 72.9 |  |
| 6 | 67 | 71.7 | 80.9 | 73.3 | 79.1 | 72.9 | 89.6 | 78.6 | 73.4 | 36.0 |  |
| 6 | 68 | 70.6 | 79.2 | 71.2 | 77.6 | 72.4 | 86.3 | 80.3 | 71.8 | 31.5 |  |
| 6 | 69 | 68.7 | 78.4 | 74.1 | 75.3 | 71.3 | 86.3 | 79.9 | 70.4 | 33.8 |  |
| 6 | 70 | 64.1 | 76.5 | 68.8 | 72.4 | 67.9 | 89.4 | 77.3 | 65.7 | 68.0 |  |
| 6 | 71 | 62.0 | 79.9 | 67.2 | 73.2 | 67.2 | 89.7 | 77.5 | 67.4 | 81.0 |  |
| 6 | 72 | 54.6 | 79.0 | 66.5 | 71.2 | 64.9 | 95.5 | 55.9 | 59.6 | 184.7 |  |
| 7 | 0 | 71.5 | 80.8 | 78.0 | 81.1 | 73.8 | 85.1 | 75.8 | 74.9 | 20.2 |  |
| 7 | 1 | 68.2 | 79.8 | 74.9 | 79.5 | 71.4 | 87.2 | 79.3 | 69.3 | 41.5 |  |
| 7 | 2 | 73.6 | 79.1 | 75.5 | 76.9 | 75.5 | 90.1 | 81.7 | 73.0 | 31.4 |  |
| 7 | 3 | 74.3 | 80.4 | 76.9 | 80.0 | 75.9 | 86.5 | 74.7 | 77.7 | 16.0 |  |
| 7 | 4 | 64.8 | 78.0 | 70.2 | 74.5 | 68.8 | 85.6 | 76.1 | 67.6 | 45.5 |  |
| 7 | 5 | 70.9 | 80.2 | 77.2 | 81.1 | 73.5 | 83.4 | 77.2 | 73.1 | 19.0 |  |
| 7 | 6 | 75.5 | 79.3 | 78.0 | 77.8 | 76.6 | 86.0 | 80.7 | 76.2 | 11.3 |  |
| 7 | 7 | 73.2 | 80.4 | 78.0 | 81.8 | 75.3 | 86.7 | 79.9 | 77.1 | 17.5 |  |
| 7 | 8 | 71.5 | 80.2 | 77.6 | 80.2 | 74.0 | 82.2 | 75.5 | 77.4 | 12.5 |  |
| 7 | 9 | 70.9 | 75.0 | 71.0 | 73.8 | 72.8 | 87.0 | 76.8 | 72.0 | 27.7 |  |
| 7 | 10 | 59.7 | 72.5 | 63.0 | 68.4 | 64.8 | 92.5 | 67.1 | 61.7 | 109.1 |  |
| 7 | 11 | 63.5 | 77.0 | 68.4 | 73.1 | 67.7 | 89.9 | 76.1 | 72.5 | 64.2 |  |
| 7 | 12 | 66.0 | 77.3 | 68.9 | 74.2 | 68.8 | 90.3 | 71.3 | 68.0 | 61.4 |  |
| 7 | 13 | 62.9 | 75.9 | 62.7 | 70.1 | 66.7 | 86.6 | 72.7 | 61.3 | 72.5 |  |
| 7 | 14 | 68.4 | 76.5 | 71.1 | 77.0 | 70.7 | 79.7 | 71.4 | 67.0 | 20.2 |  |
| 7 | 15 | 70.4 | 80.8 | 72.4 | 77.8 | 73.7 | 90.1 | 73.5 | 69.4 | 46.1 |  |
| 7 | 16 | 67.6 | 76.5 | 72.4 | 77.7 | 70.3 | 89.9 | 70.9 | 68.1 | 53.5 |  |
| 7 | 17 | 68.6 | 79.2 | 71.6 | 79.0 | 72.9 | 89.9 | 71.5 | 71.7 | 47.6 |  |
| 7 | 18 | 67.9 | 77.5 | 71.8 | 77.9 | 71.3 | 91.0 | 71.4 | 75.6 | 50.8 |  |
| 7 | 19 | 74.3 | 81.5 | 75.8 | 82.6 | 76.4 | 88.2 | 82.8 | 79.0 | 21.1 |  |
| 7 | 20 | 77.7 | 84.8 | 77.8 | 86.2 | 79.0 | 91.2 | 85.4 | 80.8 | 23.2 |  |
| 7 | 21 | 74.0 | 84.0 | 77.5 | 85.6 | 76.1 | 91.0 | 81.4 | 75.1 | 35.6 |  |
| 7 | 22 | 78.7 | 80.5 | 80.0 | 82.0 | 79.3 | 84.6 | 85.7 | 85.9 | 8.5 |  |
| 7 | 23 | 79.1 | 75.8 | 80.0 | 78.0 | 77.9 | 82.4 | 73.7 | 73.6 | 9.4 |  |
| 7 | 24 | 67.6 | 78.8 | 75.5 | 80.0 | 71.5 | 85.9 | 78.5 | 75.0 | 31.1 |  |
| 7 | 25 | 67.3 | 77.8 | 69.8 | 79.3 | 71.3 | 85.7 | 79.8 | 73.9 | 37.5 |  |
| 7 | 26 | 62.2 | 79.2 | 63.6 | 79.9 | 68.5 | 92.1 | 78.2 | 71.1 | 99.0 |  |
| 7 | 27 | 71.2 | 81.0 | 66.1 | 80.3 | 73.2 | 93.3 | 79.9 | 69.1 | 75.4 |  |
| 7 | 28 | 73.3 | 79.6 | 70.1 | 78.2 | 74.1 | 89.2 | 71.0 | 74.8 | 37.7 |  |
| 7 | 29 | 65.9 | 75.6 | 65.6 | 75.2 | 67.8 | 86.2 | 73.5 | 73.6 | 45.5 |  |
| 7 | 30 | 70.0 | 81.8 | 73.7 | 82.2 | 74.3 | 86.0 | 84.6 | 76.0 | 33.9 |  |
| 7 | 31 | 68.0 | 83.8 | 77.7 | 84.8 | 73.1 | 87.0 | 81.1 | 69.3 | 53.2 |  |
| 7 | 32 | 66.7 | 84.9 | 76.3 | 84.9 | 71.3 | 92.6 | 79.5 | 69.0 | 81.0 |  |
| 7 | 33 | 72.3 | 80.8 | 79.1 | 82.4 | 75.0 | 87.1 | 78.9 | 76.6 | 21.1 |  |
| 7 | 34 | 74.8 | 80.1 | 76.5 | 80.6 | 76.7 | 84.0 | 83.4 | 82.8 | 12.3 |  |
| 7 | 35 | 69.6 | 82.0 | 77.3 | 80.9 | 73.0 | 86.5 | 81.1 | 79.9 | 28.8 |  |
| 7 | 36 | 65.3 | 82.0 | 71.6 | 81.7 | 70.0 | 90.3 | 72.4 | 75.8 | 65.2 |  |
| 7 | 37 | 72.2 | 82.0 | 77.3 | 81.9 | 74.2 | 88.5 | 76.4 | 78.2 | 26.8 |  |
| 7 | 38 | 75.2 | 83.6 | 80.0 | 83.5 | 78.5 | 89.9 | 80.5 | 83.4 | 19.2 |  |
| 7 | 39 | 77.8 | 82.0 | 79.3 | 83.1 | 79.2 | 89.1 | 85.5 | 81.4 | 13.9 |  |
| 7 | 40 | 69.6 | 82.9 | 77.3 | 82.0 | 73.7 | 88.9 | 77.7 | 75.5 | 36.4 |  |
| 7 | 41 | 71.2 | 83.1 | 70.1 | 81.9 | 73.9 | 90.5 | 78.0 | 74.2 | 48.2 |  |
| 7 | 42 | 75.1 | 81.7 | 79.4 | 82.2 | 77.8 | 84.7 | 74.4 | 76.4 | 13.5 |  |
| 7 | 43 | 67.5 | 82.9 | 74.6 | 80.5 | 73.4 | 87.8 | 75.3 | 72.8 | 41.8 |  |
| 7 | 44 | 71.3 | 80.1 | 75.0 | 79.9 | 73.5 | 83.5 | 78.2 | 72.6 | 18.5 |  |
| 7 | 45 | 66.3 | 79.2 | 75.2 | 80.4 | 70.9 | 86.2 | 71.2 | 70.3 | 43.2 |  |
| 7 | 46 | 67.5 | 77.2 | 72.2 | 75.9 | 70.6 | 84.8 | 75.3 | 72.8 | 26.9 |  |
| 7 | 47 | 61.2 | 78.4 | 68.4 | 74.4 | 66.2 | 86.7 | 81.7 | 72.0 | 71.4 |  |
| 7 | 48 | 60.9 | 81.1 | 64.7 | 72.2 | 67.5 | 91.8 | 70.8 | 64.4 | 104.4 |  |
| 7 | 49 | 67.4 | 79.1 | 71.4 | 77.8 | 71.5 | 88.8 | 76.2 | 66.0 | 54.4 |  |
| 7 | 50 | 67.9 | 79.1 | 70.6 | 76.1 | 71.4 | 85.9 | 76.4 | 67.3 | 39.7 |  |
| 7 | 51 | 67.6 | 79.0 | 75.0 | 77.1 | 72.2 | 86.2 | 68.6 | 71.1 | 37.7 |  |
| 7 | 52 | 61.9 | 77.1 | 66.9 | 71.1 | 68.2 | 90.4 | 80.3 | 65.4 | 88.1 |  |
| 7 | 53 | 60.8 | 76.4 | 64.5 | 68.2 | 66.4 | 94.4 | 67.6 | 62.6 | 118.6 |  |
| 7 | 54 | 66.7 | 79.2 | 72.5 | 76.2 | 70.2 | 85.4 | 78.6 | 72.8 | 34.6 |  |
| 7 | 55 | 70.3 | 79.2 | 76.5 | 79.6 | 73.0 | 85.5 | 69.7 | 73.8 | 28.8 |  |
| 7 | 56 | 70.0 | 79.4 | 75.9 | 78.8 | 72.4 | 85.6 | 76.2 | 76.5 | 22.1 |  |
| 7 | 57 | 62.3 | 79.2 | 66.5 | 71.3 | 68.7 | 91.6 | 82.2 | 64.8 | 102.3 |  |
| 7 | 58 | 67.3 | 80.3 | 71.7 | 78.3 | 71.4 | 90.8 | 84.7 | 69.4 | 67.5 |  |
| 7 | 59 | 60.9 | 79.0 | 66.5 | 72.0 | 65.6 | 88.6 | 83.0 | 66.6 | 94.5 |  |
| 7 | 60 | 68.9 | 76.9 | 72.7 | 76.3 | 71.0 | 87.5 | 74.0 | 72.2 | 32.7 |  |
| 8 | 0 | 71.0 | 79.4 | 74.6 | 75.1 | 73.2 | 85.7 | 82.5 | 74.8 | 25.2 |  |
| 8 | 1 | 70.8 | 79.9 | 76.3 | 77.6 | 72.4 | 86.5 | 81.5 | 73.8 | 26.7 |  |
| 8 | 2 | 62.4 | 74.0 | 67.2 | 71.1 | 66.0 | 91.7 | 74.1 | 67.4 | 81.4 |  |
| 8 | 3 | 74.8 | 78.7 | 78.4 | 80.0 | 75.7 | 83.3 | 76.0 | 79.1 | 7.5 |  |
| 8 | 4 | 75.2 | 80.1 | 76.2 | 79.3 | 75.8 | 86.0 | 79.0 | 73.3 | 15.5 |  |
| 8 | 5 | 67.8 | 79.6 | 73.4 | 78.2 | 70.1 | 86.7 | 82.0 | 70.6 | 43.6 |  |
| 8 | 6 | 64.6 | 74.4 | 67.3 | 68.5 | 68.5 | 91.6 | 84.4 | 68.9 | 90.9 |  |
| 8 | 7 | 72.0 | 77.3 | 78.3 | 78.1 | 74.0 | 85.2 | 82.9 | 76.6 | 18.4 |  |
| 8 | 8 | 73.9 | 82.1 | 77.1 | 83.0 | 76.5 | 87.8 | 77.6 | 77.1 | 20.6 |  |
| 8 | 9 | 72.5 | 79.1 | 75.2 | 79.5 | 74.9 | 86.9 | 71.4 | 75.5 | 24.2 |  |
| 8 | 10 | 73.5 | 78.2 | 74.8 | 78.4 | 75.7 | 87.6 | 72.0 | 74.9 | 23.4 |  |
| 8 | 11 | 72.7 | 78.7 | 73.6 | 77.7 | 74.8 | 86.8 | 73.7 | 72.7 | 22.6 |  |
| 8 | 12 | 74.1 | 78.6 | 76.6 | 77.7 | 75.5 | 85.4 | 72.2 | 77.4 | 15.4 |  |
| 8 | 13 | 78.8 | 80.3 | 82.1 | 82.1 | 78.5 | 88.4 | 78.2 | 82.4 | 11.1 |  |
| 8 | 14 | 72.9 | 78.8 | 77.1 | 79.6 | 75.1 | 85.8 | 76.4 | 78.6 | 14.7 |  |
| 8 | 15 | 76.6 | 78.8 | 78.2 | 78.4 | 76.0 | 85.7 | 72.0 | 78.1 | 14.7 |  |
| 8 | 16 | 72.8 | 76.7 | 74.9 | 76.7 | 75.1 | 83.3 | 77.8 | 77.0 | 9.4 |  |
| 8 | 17 | 73.4 | 74.5 | 74.3 | 73.2 | 74.3 | 86.0 | 75.7 | 73.3 | 18.4 |  |
| 8 | 18 | 72.3 | 77.2 | 70.0 | 76.6 | 73.6 | 85.5 | 72.2 | 71.0 | 25.2 |  |
| 8 | 19 | 71.0 | 80.2 | 69.3 | 78.9 | 74.1 | 89.4 | 67.5 | 63.6 | 68.9 |  |
| 8 | 20 | 68.5 | 77.3 | 67.1 | 70.1 | 68.8 | 88.2 | 65.8 | 66.1 | 58.9 |  |
| 8 | 21 | 68.1 | 74.7 | 67.1 | 71.2 | 70.1 | 86.5 | 70.6 | 65.6 | 43.3 |  |
| 8 | 22 | 74.4 | 78.7 | 78.0 | 77.5 | 75.7 | 88.9 | 70.5 | 78.4 | 27.6 |  |
| 8 | 23 | 77.5 | 81.6 | 74.7 | 81.3 | 78.2 | 91.0 | 75.5 | 73.8 | 31.0 |  |
| 8 | 24 | 81.6 | 81.8 | 77.3 | 78.9 | 80.2 | 90.4 | 72.1 | 70.7 | 37.8 |  |
| 8 | 25 | 81.1 | 82.3 | 78.4 | 81.5 | 83.1 | 92.8 | 76.4 | 77.6 | 25.9 |  |
| 8 | 26 | 76.8 | 79.4 | 74.0 | 76.8 | 78.5 | 94.0 | 75.7 | 69.6 | 50.1 |  |
| 8 | 27 | 80.5 | 79.9 | 81.5 | 81.8 | 80.2 | 87.5 | 79.6 | 75.9 | 10.4 |  |
| 8 | 28 | 78.1 | 78.0 | 78.2 | 82.3 | 78.9 | 89.9 | 73.5 | 75.7 | 24.5 |  |
| 8 | 29 | 75.4 | 86.2 | 72.9 | 85.8 | 79.4 | 94.9 | 77.4 | 72.2 | 61.8 |  |
| 8 | 30 | 77.2 | 81.9 | 78.1 | 80.9 | 79.2 | 87.5 | 85.8 | 79.6 | 13.3 |  |
| 8 | 31 | 78.7 | 80.4 | 79.9 | 81.0 | 79.5 | 87.5 | 84.7 | 83.0 | 9.1 |  |
| 8 | 32 | 81.2 | 80.3 | 82.3 | 81.3 | 81.1 | 87.8 | 80.8 | 80.7 | 5.9 |  |
| 8 | 33 | 81.4 | 84.7 | 83.3 | 85.1 | 84.7 | 92.1 | 80.8 | 81.7 | 12.8 |  |
| 8 | 34 | 81.5 | 82.2 | 81.5 | 82.0 | 82.3 | 89.7 | 76.3 | 83.3 | 13.3 |  |
| 8 | 35 | 78.9 | 82.7 | 76.2 | 82.7 | 82.1 | 91.1 | 79.1 | 74.1 | 26.8 |  |
| 8 | 36 | 77.0 | 81.0 | 80.1 | 81.4 | 79.4 | 88.3 | 83.1 | 76.4 | 13.9 |  |
| 8 | 37 | 75.1 | 83.7 | 79.9 | 83.6 | 79.2 | 89.3 | 77.1 | 70.1 | 34.7 |  |
| 8 | 38 | 72.8 | 80.8 | 74.8 | 81.7 | 75.3 | 87.7 | 83.3 | 69.5 | 37.3 |  |
| 8 | 39 | 69.5 | 78.5 | 73.1 | 77.4 | 72.9 | 88.9 | 72.5 | 65.8 | 48.6 |  |
| 8 | 40 | 68.8 | 80.5 | 71.6 | 76.4 | 72.1 | 93.1 | 75.1 | 67.0 | 68.8 |  |
| 8 | 41 | 73.6 | 82.5 | 76.1 | 82.7 | 75.4 | 91.9 | 73.5 | 72.0 | 45.8 |  |
| 8 | 42 | 77.6 | 82.8 | 82.2 | 84.3 | 79.5 | 88.1 | 76.6 | 80.0 | 14.3 |  |
| 8 | 43 | 71.4 | 77.8 | 76.7 | 78.1 | 73.9 | 84.3 | 77.0 | 74.8 | 14.5 |  |
| 8 | 44 | 72.7 | 82.9 | 77.6 | 82.8 | 75.1 | 87.9 | 77.1 | 75.3 | 26.0 |  |
| 8 | 45 | 74.3 | 78.8 | 74.8 | 79.4 | 76.0 | 88.9 | 69.8 | 72.7 | 33.5 |  |
| 8 | 46 | 66.1 | 73.0 | 69.4 | 72.8 | 69.2 | 95.1 | 69.8 | 72.3 | 82.0 |  |
| 8 | 47 | 68.2 | 77.1 | 70.2 | 76.0 | 71.9 | 93.7 | 71.2 | 68.1 | 70.8 |  |
| 8 | 48 | 62.4 | 73.5 | 66.5 | 70.5 | 68.4 | 91.5 | 73.7 | 64.5 | 82.2 |  |
| 8 | 49 | 74.3 | 78.7 | 75.6 | 77.3 | 76.3 | 87.7 | 74.6 | 73.8 | 20.3 |  |
| 8 | 50 | 70.0 | 78.9 | 77.9 | 80.3 | 74.1 | 87.0 | 71.0 | 73.8 | 31.1 |  |
| 8 | 51 | 73.9 | 80.5 | 76.5 | 79.6 | 76.3 | 87.9 | 78.4 | 73.9 | 20.7 |  |
| 8 | 52 | 72.7 | 79.1 | 74.7 | 78.7 | 74.9 | 84.7 | 73.8 | 75.3 | 15.4 |  |
| 8 | 53 | 73.5 | 81.6 | 77.7 | 81.8 | 75.0 | 84.6 | 75.9 | 76.9 | 15.1 |  |
| 8 | 54 | 71.0 | 81.1 | 77.1 | 81.6 | 72.9 | 84.1 | 83.9 | 77.9 | 23.9 |  |
| 8 | 55 | 67.7 | 77.8 | 72.3 | 74.8 | 70.0 | 88.1 | 83.4 | 70.4 | 50.7 |  |
| 8 | 56 | 67.5 | 77.1 | 69.3 | 74.8 | 71.1 | 86.7 | 77.9 | 67.2 | 43.6 |  |
| 8 | 57 | 70.9 | 78.8 | 73.8 | 77.5 | 73.4 | 86.0 | 79.5 | 72.6 | 24.1 |  |
| 8 | 58 | 66.5 | 77.4 | 69.0 | 73.9 | 69.0 | 89.5 | 78.5 | 69.7 | 56.7 |  |
| 8 | 59 | 72.8 | 80.6 | 76.2 | 79.2 | 74.9 | 88.1 | 78.7 | 76.9 | 21.4 |  |
| 8 | 60 | 68.2 | 78.4 | 72.5 | 77.0 | 70.7 | 84.8 | 75.8 | 73.1 | 26.8 |  |
| 8 | 61 | 66.2 | 80.4 | 72.9 | 76.0 | 71.0 | 87.7 | 80.0 | 71.0 | 46.6 |  |
| 9 | 0 | 70.2 | 81.0 | 76.5 | 81.3 | 72.7 | 86.4 | 77.0 | 75.6 | 26.7 |  |
| 9 | 1 | 72.7 | 80.4 | 77.4 | 79.5 | 74.5 | 86.4 | 75.7 | 75.0 | 19.0 |  |
| 9 | 2 | 68.3 | 76.8 | 73.2 | 75.4 | 71.5 | 87.0 | 76.2 | 72.3 | 30.7 |  |
| 9 | 3 | 71.7 | 80.2 | 76.8 | 81.9 | 74.9 | 85.9 | 82.8 | 76.2 | 22.1 |  |
| 9 | 4 | 74.8 | 80.6 | 78.0 | 82.4 | 76.7 | 87.9 | 77.7 | 77.1 | 17.2 |  |
| 9 | 5 | 74.2 | 81.4 | 77.7 | 81.4 | 75.6 | 88.8 | 75.3 | 78.9 | 22.6 |  |
| 9 | 6 | 69.5 | 79.7 | 73.0 | 77.8 | 73.0 | 88.0 | 78.4 | 71.5 | 34.9 |  |
| 9 | 7 | 71.9 | 80.3 | 74.3 | 79.6 | 75.7 | 89.2 | 78.7 | 75.6 | 27.9 |  |
| 9 | 8 | 73.5 | 81.4 | 77.4 | 80.8 | 76.2 | 84.6 | 78.9 | 76.3 | 12.3 |  |
| 9 | 9 | 70.4 | 82.1 | 78.2 | 81.8 | 73.9 | 88.4 | 78.9 | 76.1 | 30.5 |  |
| 9 | 10 | 70.9 | 82.4 | 77.4 | 83.2 | 75.4 | 90.3 | 78.8 | 76.3 | 35.1 |  |
| 9 | 11 | 70.9 | 74.0 | 72.2 | 72.2 | 72.5 | 86.8 | 73.5 | 69.5 | 28.9 |  |
| 9 | 12 | 72.7 | 78.7 | 76.5 | 78.6 | 74.6 | 83.4 | 75.5 | 75.7 | 10.6 |  |
| 9 | 13 | 71.8 | 84.3 | 70.0 | 84.9 | 78.3 | 91.1 | 70.3 | 56.4 | 121.5 |  |
| 9 | 14 | 74.3 | 79.9 | 73.1 | 81.7 | 75.9 | 84.5 | 76.5 | 77.4 | 15.0 |  |
| 9 | 15 | 66.9 | 79.9 | 66.3 | 77.1 | 72.9 | 93.3 | 63.3 | 65.1 | 102.3 |  |
| 9 | 16 | 60.3 | 76.7 | 60.5 | 72.6 | 67.9 | 97.0 | 68.4 | 59.5 | 154.1 |  |
| 9 | 17 | 72.6 | 78.6 | 73.8 | 78.4 | 76.3 | 97.5 | 73.3 | 74.8 | 66.2 |  |
| 9 | 18 | 64.6 | 79.1 | 65.2 | 79.0 | 70.0 | 95.2 | 64.7 | 65.8 | 118.4 |  |
| 9 | 19 | 66.4 | 77.6 | 69.0 | 76.9 | 70.3 | 91.9 | 73.7 | 70.0 | 64.9 |  |
| 9 | 20 | 70.3 | 80.6 | 70.6 | 79.8 | 73.6 | 86.0 | 75.4 | 73.2 | 30.0 |  |
| 9 | 21 | 74.8 | 85.7 | 75.2 | 86.4 | 78.0 | 90.9 | 87.8 | 80.1 | 37.3 |  |
| 9 | 22 | 70.4 | 83.7 | 73.5 | 83.8 | 73.3 | 88.1 | 76.2 | 76.9 | 39.0 |  |
| 9 | 23 | 64.1 | 85.3 | 68.6 | 86.3 | 71.7 | 92.6 | 71.4 | 66.2 | 114.2 |  |
| 9 | 24 | 74.7 | 83.4 | 76.3 | 83.5 | 78.1 | 89.8 | 81.5 | 77.8 | 24.0 |  |
| 9 | 25 | 70.8 | 83.6 | 71.0 | 82.6 | 75.7 | 88.4 | 73.0 | 74.3 | 43.1 |  |
| 9 | 26 | 68.1 | 83.6 | 73.9 | 81.1 | 75.7 | 88.2 | 77.3 | 77.1 | 38.1 |  |
| 9 | 27 | 71.0 | 86.2 | 66.4 | 83.1 | 75.3 | 93.8 | 66.6 | 64.2 | 116.7 |  |
| 9 | 28 | 64.4 | 86.3 | 60.3 | 84.3 | 69.7 | 93.0 | 68.8 | 64.4 | 147.8 |  |
| 9 | 29 | 71.7 | 80.9 | 71.0 | 81.2 | 74.5 | 90.9 | 80.1 | 78.1 | 41.1 |  |
| 9 | 30 | 76.7 | 77.1 | 79.9 | 76.5 | 77.6 | 92.6 | 70.8 | 68.9 | 50.5 |  |
| 9 | 31 | 69.0 | 77.6 | 74.4 | 75.9 | 74.9 | 95.8 | 64.4 | 66.3 | 94.9 |  |
| 9 | 32 | 66.3 | 80.4 | 73.4 | 73.0 | 72.4 | 98.9 | 59.6 | 67.5 | 139.1 |  |
| 9 | 33 | 68.8 | 79.4 | 66.7 | 75.2 | 74.4 | 95.5 | 65.9 | 64.0 | 105.5 |  |
| 9 | 34 | 75.3 | 79.5 | 76.3 | 79.4 | 76.5 | 86.3 | 78.7 | 76.0 | 12.6 |  |
| 9 | 35 | 80.3 | 81.4 | 79.8 | 82.6 | 80.0 | 84.1 | 77.4 | 81.2 | 4.0 |  |
| 9 | 36 | 77.0 | 82.2 | 80.9 | 81.7 | 80.0 | 86.9 | 79.9 | 76.3 | 10.9 |  |
| 9 | 37 | 74.2 | 81.8 | 75.5 | 79.7 | 75.5 | 85.8 | 78.8 | 76.8 | 15.2 |  |
| 9 | 38 | 73.6 | 80.0 | 76.2 | 79.2 | 75.7 | 85.8 | 79.0 | 74.5 | 15.3 |  |
| 9 | 39 | 69.8 | 75.8 | 73.2 | 74.4 | 71.6 | 89.2 | 74.9 | 70.9 | 37.5 |  |
| 9 | 40 | 76.6 | 80.0 | 77.8 | 81.3 | 77.4 | 85.2 | 75.0 | 78.9 | 10.1 |  |
| 9 | 41 | 74.0 | 79.6 | 76.9 | 81.7 | 75.2 | 85.7 | 70.4 | 79.8 | 23.1 |  |
| 9 | 42 | 69.1 | 78.6 | 73.1 | 77.2 | 73.1 | 87.5 | 78.1 | 74.3 | 30.2 |  |
| 9 | 43 | 70.9 | 79.5 | 76.7 | 77.0 | 73.2 | 89.2 | 70.6 | 74.0 | 36.3 |  |
| 9 | 44 | 71.4 | 80.7 | 76.9 | 80.3 | 73.4 | 87.8 | 80.3 | 76.4 | 26.0 |  |
| 9 | 45 | 66.9 | 76.2 | 68.5 | 71.3 | 70.6 | 89.0 | 74.6 | 68.1 | 51.4 |  |
| 9 | 46 | 72.4 | 81.6 | 77.8 | 81.3 | 74.2 | 88.4 | 82.9 | 75.2 | 28.3 |  |
| 9 | 47 | 71.7 | 80.8 | 77.2 | 80.7 | 74.0 | 86.4 | 81.4 | 75.6 | 22.6 |  |
| 9 | 48 | 72.5 | 79.6 | 75.2 | 78.3 | 74.0 | 86.8 | 79.9 | 76.4 | 20.1 |  |
| 9 | 49 | 66.4 | 78.0 | 74.3 | 77.2 | 70.2 | 91.7 | 72.2 | 71.9 | 58.4 |  |
| 9 | 50 | 68.3 | 80.3 | 71.5 | 78.4 | 70.7 | 84.7 | 76.6 | 71.0 | 32.7 |  |
| 9 | 51 | 65.5 | 76.8 | 70.1 | 73.8 | 67.2 | 88.2 | 72.8 | 68.9 | 51.7 |  |
| 9 | 52 | 68.9 | 78.4 | 74.4 | 77.1 | 70.4 | 86.3 | 76.6 | 72.8 | 29.6 |  |
| 9 | 53 | 77.3 | 80.0 | 79.1 | 81.3 | 77.5 | 84.3 | 74.2 | 81.6 | 9.7 |  |
| 9 | 54 | 69.5 | 78.9 | 75.3 | 78.2 | 70.7 | 83.8 | 79.1 | 75.0 | 22.0 |  |
| 9 | 55 | 67.7 | 80.0 | 79.2 | 79.8 | 69.8 | 84.7 | 76.1 | 77.9 | 31.4 |  |

^a^Chro = Chromosome
