# Supplementary material for: Genetic drift, historic migration, and limited gene flow contributing to the subpopulation divergence in wild sea beet (Beta vulgaris ssp. maritima (L.) Arcang)
Source: PLoS One. 2024 Sep 6;19(9):e0308626. doi: 10.1371/journal.pone.0308626 (PMC11379190; doi:10.1371/journal.pone.0308626)
Supplement: Attachment — Submitted filename: response to reviewers.docx [file pone.0308626.s006.docx]

**Response to Academic Editor and Reviewers**

**Response to Editor:**

When submitting your revision, we need you to address these additional requirements.

1. Please ensure that your manuscript meets PLOS ONE's style requirements, including those for file naming. The PLOS ONE style templates can be found at 
<https://journals.plos.org/plosone/s/file?id=wjVg/PLOSOne_formatting_sample_main_body.pdf> and 
<https://journals.plos.org/plosone/s/file?id=ba62/PLOSOne_formatting_sample_title_authors_affiliations.pdf>

*Response: format was checked and corrected according to the guidelines.*

2. We note that the grant information you provided in the ‘Funding Information’ and ‘Financial Disclosure’ sections do not match. When you resubmit, please ensure that you provide the correct grant numbers for the awards you received for your study in the ‘Funding Information’ section.

*Response: grant numbers added.*

3. Thank you for stating the following financial disclosure: 
"USDA-ARS CRIS project No. 3060-21000-045-000D, the Beet Sugar Development Foundation (BSDF), and the Sugarbeet Research and Education Board of Minnesota and North Dakota (SBREB)."
  
Please state what role the funders took in the study.  If the funders had no role, please state: "The funders had no role in study design, data collection and analysis, decision to publish, or preparation of the manuscript." 
If this statement is not correct you must amend it as needed.

Please include this amended Role of Funder statement in your cover letter; we will change the online submission form on your behalf.

*Response: the statement is added.*

4. In the online submission form, you indicated that [All relevant data are within the manuscript and its Supporting Information files. Raw data are available per request.]. 
All PLOS journals now require all data underlying the findings described in their manuscript to be freely available to other researchers, either 1. In a public repository, 2. Within the manuscript itself, or 3. Uploaded as supplementary information.
This policy applies to all data except where public deposition would breach compliance with the protocol approved by your research ethics board. If your data cannot be made publicly available for ethical or legal reasons (e.g., public availability would compromise patient privacy), please explain your reasons on resubmission and your exemption request will be escalated for approval.

*Response: All data are available and attached, and the raw marker data is stored at NPGS-GRIN and is publicly available. This information is added in the manuscript.*

5. We note that [Figure 4 and S3] in your submission contain [map/satellite] images which may be copyrighted. All PLOS content is published under the Creative Commons Attribution License (CC BY 4.0), which means that the manuscript, images, and Supporting Information files will be freely available online, and any third party is permitted to access, download, copy, distribute, and use these materials in any way, even commercially, with proper attribution. For these reasons, we cannot publish previously copyrighted maps or satellite images created using proprietary data, such as Google software (Google Maps, Street View, and Earth). For more information, see our copyright guidelines: <http://journals.plos.org/plosone/s/licenses-and-copyright>. 
We require you to either (1) present written permission from the copyright holder to publish these figures specifically under the CC BY 4.0 license, or (2) remove the figures from your submission: 
a. You may seek permission from the original copyright holder of Figure 4 and S3 to publish the content specifically under the CC BY 4.0 license.   
We recommend that you contact the original copyright holder with the Content Permission Form (<http://journals.plos.org/plosone/s/file?id=7c09/content-permission-form.pdf>) and the following text:
“I request permission for the open-access journal PLOS ONE to publish XXX under the Creative Commons Attribution License (CCAL) CC BY 4.0 (<http://creativecommons.org/licenses/by/4.0/>). Please be aware that this license allows unrestricted use and distribution, even commercially, by third parties. Please reply and provide explicit written permission to publish XXX under a CC BY license and complete the attached form.” 
Please upload the completed Content Permission Form or other proof of granted permissions as an "Other" file with your submission. 
In the figure caption of the copyrighted figure, please include the following text: “Reprinted from [ref] under a CC BY license, with permission from [name of publisher], original copyright [original copyright year].”
 b. If you are unable to obtain permission from the original copyright holder to publish these figures under the CC BY 4.0 license or if the copyright holder’s requirements are incompatible with the CC BY 4.0 license, please either i) remove the figure or ii) supply a replacement figure that complies with the CC BY 4.0 license. Please check copyright information on all replacement figures and update the figure caption with source information. If applicable, please specify in the figure caption text when a figure is similar but not identical to the original image and is therefore for illustrative purposes only.
The following resources for replacing copyrighted map figures may be helpful: 
USGS National Map Viewer (public domain): <http://viewer.nationalmap.gov/viewer/>
The Gateway to Astronaut Photography of Earth (public domain): <http://eol.jsc.nasa.gov/sseop/clickmap/>
Maps at the CIA (public domain): <https://www.cia.gov/library/publications/the-world-factbook/index.html> and <https://www.cia.gov/library/publications/cia-maps-publications/index.html>
NASA Earth Observatory (public domain): <http://earthobservatory.nasa.gov/>
Landsat: <http://landsat.visibleearth.nasa.gov/>
USGS EROS (Earth Resources Observatory and Science (EROS) Center) (public domain): [http://eros.usgs.gov/#](http://eros.usgs.gov/)
Natural Earth (public domain): <http://www.naturalearthdata.com/>

*Response: the figures were reproduced using NASA world map (https://data.nasa.gov/), and ocean current direction was manually added according to the reference listed.*

6. Please include captions for your Supporting Information files at the end of your manuscript, and update any in-text citations to match accordingly. Please see our Supporting Information guidelines for more information: <http://journals.plos.org/plosone/s/supporting-information>.

*Response: added.*

7. Please review your reference list to ensure that it is complete and correct. If you have cited papers that have been retracted, please include the rationale for doing so in the manuscript text, or remove these references and replace them with relevant current references. Any changes to the reference list should be mentioned in the rebuttal letter that accompanies your revised manuscript. If you need to cite a retracted article, indicate the article’s retracted status in the References list and also include a citation and full reference for the retraction notice.

*Response: all references were checked.*

**Responses to Reviewers' comments:**

**Reviewer #1:**

First, I extend my gratitude to the authors for their outstanding work and well-constructed text. In this study, authors investigated 599 sea beet (Beta maritima) accessions collected from the north Atlantic Ocean and Mediterranean Sea coasts. It is a comprehensive study about the distribution and origin of sea beet and cultivated beet. The title explains the research properly, and the results are described well in details. I think the manuscript contains a good research question and explains the migration route of wild beet along the north Atlantic Ocean and Mediterranean Sea coasts. In the future, the findings will be useful to identify novel genomic regions related to resistance against abiotic/biotic stress factors in Beta vulgaris. However, I have some comments as follows. I recommend minor revisions.
Here are my comments:

- In Materials and Methods, authors did not describe the growth conditions of beet accessions.

*Response: information was added.*

- Authors should mention how many accessions they used from each of the following: Africa, Asia, northern Europe, southern Europe, western Europe, eastern Europe, and North America.

*Response: number of accessions were added for each region. The region ‘eastern Europe’ is deleted since the two lines have not good marker data and were not included in the analysis.*

-In Figures 4 and S3, why did not authors show the location of Cluster 6?

*Response: it has two reasons: 1) the cluster 6 is corresponding to cultivated beet that selected worldwide, not a specific location is assigned; and 2) B. maritima accessions in the cluster 6 are spread similarly in several other clusters with no dominant amount from a specific region, it is impossible to assign a location for those accessions.*

- That map does not show the locations correctly unless I have missed something. For example, I do not see Greece and Italy in Cluster 2 (please see discussion, line 306).

*Response: Thank you so much for pointing this out. When making the figure, we only thought to find regions and try to make them distinct, but clusters 2 and 4 are very close and their regions are largely overlapped. We thus enlarged the region of the cluster 2 to let it covers Italy and Greece, and it is more consistent with our results. Great thanks!*

- Why do you think the Cluster 2 is the center of origin for sea beet and cultivated beet? You may highlight the experimental results for clearer explanation.

*Response: Cluster 2 is the center of origin for sea beet simply because it contains accessions collected from almost all regions, the location of regions for Cluster 2 is determined based on the location that majority of the accessions were collected. Accessions in Cluster 2 but collected from the other regions are indication of they were migrated from the region we defined, indicating regions assigned to major accessions in Cluster 2 at Mediterranean Sea coast likely to be the center of sea beet. However, the sea beet accessions close to Cluster 6 that equivalent to cultivated beet were largely collected in Greece, indicating Greece might be the center of cultivated beet domesticated from sea beet.*

- In addition, each cluster (1-8) must be briefly described in the legend of figure S3.

*Response: Great thanks for this suggestion, cluster descriptions were added accordingly. We appreciate your comments to improve the manuscript.*

**Reviewer #2:**

- Correct name: Beta vulgaris subsp. maritima (L.) Arcang. Adding to the title and in the first citation of the text the authorship of the species -> Verify: [www.tropicos.org](http://www.tropicos.org) I suggest adding the botanical family name to the title or in the keywords.

*Response: Great thanks, the scientific name was corrected in the title and in the text.*

- I suggest adding the scientific name of the causal agents of the diseases mentioned in paragraph 3 of the Introduction.

*Response: the causal agents of the diseases were added.*

- I congratulate the authors on this present work, of fundamental importance for the establishment or better management of genetic improvement programs, as well as for the conservation of wild species.

*Response: great thanks and appreciate your comments for improving the manuscript.*

**Reviewer #3:**

The origin of cultivated plants and the identification of the most closely related native populations, as well as their crop wild relatives, is a highly topical subject, given its potential for the genetic improvement of crops. Following on from other work that has been carried out on sugar beet, the submitted manuscript analyses more than six hundred samples of Beta maritima from various regions of the world. The results presented are interesting and improve the knowledge of the phylogenetic relationships of the populations, particularly those occurring in the Mediterranean region.

In general, the manuscript is well-written, well-structured, and easy to understand.

Apart from a few minor suggestions/comments (listed below), there is one aspect that I would like to highlight because I think it should be corrected: some of the samples used are from areas where Beta maritima is not native, but naturalised (this is the case with the samples from North America). I think this should be mentioned/analysed throughout the text (material and methods, results and discussion, tables, etc.). This aspect can even add value to the discussion, as hypotheses can be put forward for the origin of the populations in the regions where Beta maritima is naturalised.

*Response: We agree the location of some accessions not the location they were collected, as you indicated that accessions from North America may be donated by some institution oversea. Since the number of such accessions are small and they didn’t affect the results much, we didn’t mention about them in the discussion. Actually, we just started a new project using conserved gene sequence to investigate the evolution of B. maritima accessions as well as cultivated beets, which will be better to differentiate B. maritima accessions and then narrow down region of the origin.*

Other comments:

Ln. 54 – ‘Sea beet (Beta vulgaris L. ssp. maritima Arcang., referred to as B. maritima hereafter)’ – Currently, some of the world's most important taxonomic databases (e.g. Plants of the World Online - POWO; and World Flora Online - WFO) only accept Beta vulgaris subsp. vulgaris and consider Beta vulgaris subsp. maritima as a synonym. This does not invalidate the work developed, but it can be mentioned in the text that the work carried out used the classification adopted by the US National Plant Germplasm System (NPGS).

*Response: Thanks. We made the change accordingly.*

ln. 59 – ‘Wild B. maritima populations extend along the Mediterranean basin and the Atlantic coast.’ – This description should be more detailed (see POWO) and the regions where the species is introduced should be indicated.

*Response: More details were added according to POWO, but we are not sure if it’s correct for mentioning accessions were introduced. I heard that few B. maritima accessions from California, USA might be natively collected, but no further information to indicate if it’s true.*

Ln. 76 – ‘Tehseen et al’ - Tehseen et al. The full stop is missing.

*Response: Corrected. Thanks!*

Ln. 108 to 111 – Detail the distribution (native or non-native) in the mentioned regions. In Table 1, mark (e.g. with a *) the accessions collected in regions where the taxa is not native (e.g. North America). Check the values in this table: the total is 601 and not 599 as it should be.

*Response: Detailed information was added, accordingly. It should be 599 accessions. The two accessions from eastern Europe have not good marker data and were removed from analysis later, but we forget to change the table, accordingly.*

Check the legend of Fig. 1 (ln 524) - references to A and B are missing.

*Response: Thanks for finding this. We changed the figure but forget to change the figure legends. It’s now corrected.*

In Results section, I think Fig. 3 (Ln. 208) could be made supplementary and Fig. S3 (ln. 231) be included in the main text. The latter is quite enlightening. However, its graphic presentation could be improved.

*Response: Thanks for this comment. We originally put Figure S3 in the main text, but considered the figure was published, we then move it as supplemental file. This did flag out by academic editor and the journal. We have to redo the figure but still think it’s better to put it as the supplementary.*

ln. 273 – ‘… is influenced by a combination of landscape characteristics.’ - I suggest replacing 'landscape' (which has aesthetic connotations) with environment.

*Response: Thanks. We changed it as suggested.*

Ln. 300 – In the discussion, this manuscript could also consider the results of the paper Veloso et al. 2021 (Genetic Diversity and Population Structure of Wild Beets (Beta spp.) from the Western Iberian Peninsula and the Azores and Madeira Islands) which also highlights the importance of ocean currents in species dispersal.

*Response: Thanks. The reference is cited.*

Finally, I suggest complementing the discussion by including some conservation proposals, namely the prioritisation of certain populations / regions taking into account their phylogenetic affinities and genetic diversity.

*Response: Thanks. This was added. Great thanks for your comments to improve this manuscript.*
